# Brain Connectivity Predicts Placebo Response across Chronic Pain Clinical Trials

**DOI:** 10.1371/journal.pbio.1002570

**Published:** 2016-10-27

**Authors:** Pascal Tétreault, Ali Mansour, Etienne Vachon-Presseau, Thomas J. Schnitzer, A. Vania Apkarian, Marwan N. Baliki

**Affiliations:** 1 Department of Physiology, Northwestern University, Feinberg School of Medicine, Chicago, Illinois, United States of America; 2 Department of Physical Medicine and Rehabilitation, Northwestern University, Feinberg School of Medicine, Chicago, Illinois, United States of America; 3 Department of Internal Medicine, Northwestern University, Feinberg School of Medicine, Chicago, Illinois, United States of America; 4 Department of Anesthesia, Northwestern University, Feinberg School of Medicine, Chicago, Illinois, United States of America; 5 Rehabilitation Institution of Chicago, Chicago, Illinois, United States of America; University of Colorado Boulder, UNITED STATES

## Abstract

Placebo response in the clinical trial setting is poorly understood and alleged to be driven by statistical confounds, and its biological underpinnings are questioned. Here we identified and validated that clinical placebo response is predictable from resting-state functional magnetic-resonance-imaging (fMRI) brain connectivity. This also led to discovering a brain region predicting active drug response and demonstrating the adverse effect of active drug interfering with placebo analgesia. Chronic knee osteoarthritis (OA) pain patients (*n* = 56) underwent pretreatment brain scans in two clinical trials. Study 1 (*n* = 17) was a 2-wk single-blinded placebo pill trial. Study 2 (*n* = 39) was a 3-mo double-blinded randomized trial comparing placebo pill to duloxetine. Study 3, which was conducted in additional knee OA pain patients (*n* = 42), was observational. fMRI-derived brain connectivity maps in study 1 were contrasted between placebo responders and nonresponders and compared to healthy controls (*n* = 20). Study 2 validated the primary biomarker and identified a brain region predicting drug response. In both studies, approximately half of the participants exhibited analgesia with placebo treatment. In study 1, right midfrontal gyrus connectivity best identified placebo responders. In study 2, the same measure identified placebo responders (95% correct) and predicted the magnitude of placebo’s effectiveness. By subtracting away linearly modeled placebo analgesia from duloxetine response, we uncovered in 6/19 participants a tendency of duloxetine enhancing predicted placebo response, while in another 6/19, we uncovered a tendency for duloxetine to diminish it. Moreover, the approach led to discovering that right parahippocampus gyrus connectivity predicts drug analgesia after correcting for modeled placebo-related analgesia. Our evidence is consistent with clinical placebo response having biological underpinnings and shows that the method can also reveal that active treatment in some patients diminishes modeled placebo-related analgesia.

**Trial Registration** ClinicalTrials.gov NCT02903238

ClinicalTrials.gov NCT01558700

## Abstract 2

The biological basis of the placebo effect is poorly understood. This study shows that connectivity of the prefrontal cortex predicts the propensity for placebo response in chronic pain patients.

## Author Summary

Placebo response is extensively studied in healthy subjects and for experimental manipulations. However, in the clinical setting it has been primarily relegated to statistical confounds. Here, for the first time we examine the predictability of future placebo response in the clinical setting in patients with chronic osteoarthritis pain. We examine resting-state functional magnetic-resonance-imaging (fMRI) brain connectivity prior to the start of the clinical trial and in the setting of neutral instructions regarding treatment. Our results show that clinical placebo pill ingestion shows stronger analgesia than no treatment and is predictable from resting state blood-oxygen-level-dependent (BOLD) fMRI, and right midfrontal gyrus degree count (extent of functional connectivity) identifies placebo pill responders in one trial and can be validated (95% correct) in the placebo group of a second trial, but not in the active drug treatment (duloxetine) group. By modeling the expected placebo response in subjects receiving active drug treatment, we uncover a placebo-corrected drug response predictive brain signal and show that in some subjects the active drug tends to enhance predicted placebo response, while in others it interferes with it. Together, these results provide some evidence for clinical placebo being predetermined by brain biology and show that brain imaging may also identify a placebo-corrected prediction of response to active treatment.

## Introduction

Positive medical responses to placebo treatments are a well-recognized phenomenon observed in many pathologies, with a higher prevalence for neurological and painful conditions [1,2]. Placebo analgesia is observed ubiquitously in pain treatment trials, especially in chronic pain populations, in which it often exhibits sustained effectiveness rivaling in magnitude that of the active treatment [3–5]. Yet, interpretation of placebo response in clinical observations remains questionable because of experimental design weaknesses, as repeatedly pointed out in the past [6–8]. So far, brain markers for placebo pain relief have been mostly studied for acute pain in the healthy population [9–13], in which individual subject responses seem highly variable and prone to a multiplicity of influences [12]. Accumulating evidence indicates that neuroimaging findings of certain brain measures (neuromarkers or neural signatures) can predict acute pain perception [14], development of chronic pain [15], future learning [16], intelligence [17], and responses to pharmacological or behavioral treatments [18]. Because placebo response is believed to be driven by central nervous system mechanisms involved in expectation and inference about previous experience [11,12,19–22], it is reasonable to presume that specific brain measures may predispose individuals to a placebo response. Recent neuroimaging [13,21,23] and genetic polymorphism [24] studies show results consistent with this hypothesis.

Given the enormous societal toll of chronic pain [25], being able to predict placebo responders in a chronic pain population could both help the design of personalized medicine and enhance the success of clinical trials [6,26]. In patients with chronic knee osteoarthritis pain (OA), we used resting-state functional magnetic resonance imaging (rs-fMRI) combined with a standard clinical trial design to derive an unbiased brain-based neurological marker to predict analgesia associated with placebo treatment. We hypothesized that brain regional network information sharing (functional connectivity) properties should identify the placebo response. We reasoned that if we could uncover a brain marker prior to the start of the trials that forecasts how individual subjects will perform during these trials, then we could conclude that clinical placebo response, at least for sugar pill ingestion, is a predetermined brain process controlling the placebo response and with biological underpinnings useful in clinical decision making. After identifying and validating a brain connectivity-based placebo predictor derived from rs-fMRI scans before the start of treatment, we linearly modeled a predicted placebo response and applied it to the active drug treatment portion of the study. With this approach, we identified a brain region where functional connectivity was predictive of drug treatment response presumed to be minimally dependent on the influence of placebo. This procedure in turn uncovered the adverse effect of active treatment interfering with predicted placebo response.

## Results

**Fig 1:**
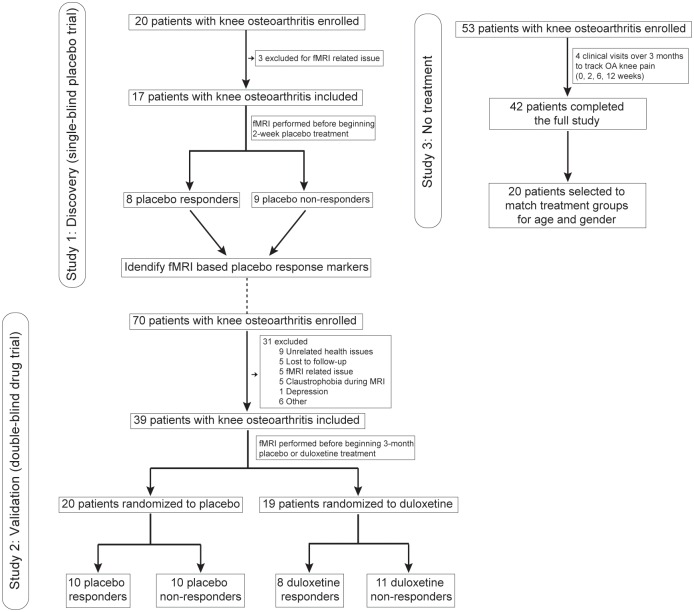
Flow diagram summarizes overall experimental design, OA patients entering and completing each of the three studies, and participant subgroupings based on treatment and treatment effects. Study 1 was analyzed to discover brain connectivity predicting placebo response. All patients received only placebo pills. Study 2 was used to validate the results from Study 1 and also to examine how the active treatment was related to placebo response. Study 2 was a double-blind randomized clinical trial. Study 3 was an observation-only trial in which no treatment was provided. Groupings and dropout causes are indicated. fMRI, functional magnetic resonance imaging.

We sought to identify a neurological signature for analgesia associated with placebo treatment in an unbiased setting. To this end, all brain imaging scans were done before the start of the clinical trials, and we studied rs-fMRI to interrogate and capture ongoing baseline brain information sharing properties (functional connectivity-based degree counts; the degree count for a given brain location is the number of voxels zero-lag correlated with this brain location above a threshold, where threshold is subject specific and determined to maintain a fixed whole-brain connectivity density; see Methods) in the absence of any manipulations of expectancies for pain relief. This design enables posing the question: to what extent is clinical placebo response predetermined by initial brain physiology and thus predictable? Placebo- or drug treatment-associated analgesia was tracked in knee OA pain patients in the setting of standard clinical trials designed to assess the efficacy of analgesics. All participating patients received the same standard instructions—i.e., that they would receive either placebo or a standard-of-care active treatment. Study 1 (*n* = 17; see Fig 1 and S1 Table for study design and demographics) was single blinded (subjects were unaware of whether they were receiving active treatment or placebo); all participants received placebo for 2 wk and underwent a washout period of no treatment for another 2 wk. The washout part of the study tested the extent of reversibility of the placebo response. Study 2 (*n* = 39) was a double-blind randomized trial in which OA subjects were randomized to either placebo or duloxetine treatment for 3 mo. In addition, in study 3 (*n* = 42) we tracked changes in knee OA pain over a 3-mo period (see S2 Table). These participants did not undergo brain scans, were not given any new treatments, and were used as a no-treatment comparison to study 1 and study 2 pain outcomes. Separate experimenters collected study 1, study 2, and study 3 data. Participants in study 1 and 2 were categorized into placebo responders and nonresponders based on a fixed threshold for pain relief (visual analog scale [VAS] score) over treatment duration. Whole-brain degree count maps from study 1 were contrasted between placebo responders and nonresponders to discover brain connectivity properties that predict placebo response propensity. Healthy subjects’ (*n* = 20) brain degree counts were used to test for correspondences between OA and healthy subjects for the primary outcome derived from study 1. Given that the identified brain regions were based on categorization of participants, we also tested whether these brain parameters of interest reflected the continuous magnitude of change in multiple pain scales (magnitude of % analgesia), which would establish the involvement of the brain regions in future placebo-related analgesia independently from the specific choice of pain threshold. Study 2 was used to validate the primary outcome derived from study 1, to explore placebo and active drug interactions, and to identify a brain region predicting active drug response.

### Placebo Pill Analgesia Effect Size

**Fig 2:**
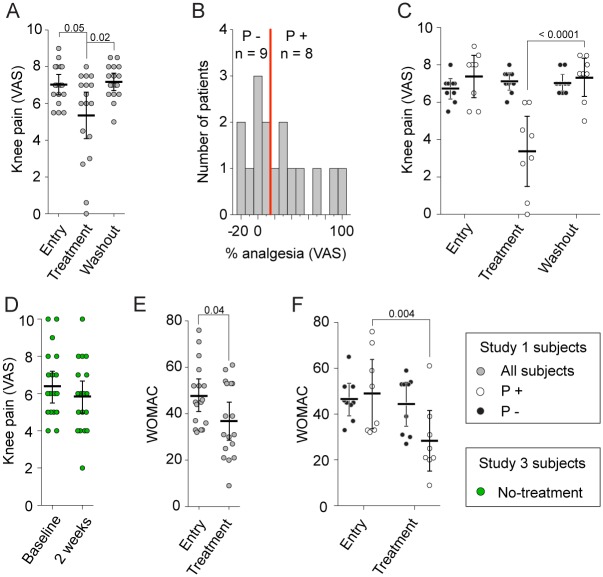
Placebo response in the single-blind placebo treatment, study 1. (A) In patients with knee OA pain (study 1), there was significant pain relief (visual analog scale [VAS], 0–10) with a 2-wk placebo treatment, which reversed to entry-level knee pain following a 2-wk placebo washout (repeated-measures analysis of variance [ANOVA], F_2,32_ = 6.8, *p* = 0.003). (B) Distribution for % analgesia (change in knee pain in VAS units from entry to 2-wk placebo treatment). The group was subdivided into placebo responders (P +) (≥20% analgesia over the 2-wk placebo treatment) and nonresponders (P −). (C) Knee OA pain shown separately for placebo responders (white) and nonresponders (black). As defined, the only decrease in pain is seen in placebo responders, after 2-wk placebo treatment. (D) Twenty knee OA pain patients (study 3), matched for age, gender, and knee VAS pain at baseline, followed over 2 wk with no treatment. There was no within-group change in knee pain over 2 wk. (E) Improvement in overall OA function (Western Ontario and McMaster Universities Osteoarthritis Index [WOMAC] scale) was observed with 2-wk placebo treatment (F_1,16_ = 6.21, *p* = 0.024). (F) The improvement was limited to placebo responders. Error bars are 95% confidence intervals (CIs). The illustrated *p*-values are post hoc comparisons that were statistically significant.

In study 1, 2 wk of placebo treatment was associated with a significant decrease in knee OA pain, with both VAS and Western Ontario and McMaster Universities Osteoarthritis Index (WOMAC) scores, across all 17 subjects (Fig 2A and 2E). At the end of the 2-wk placebo treatment period, 8/17 (47%) of participants were classified as placebo responders (based on individual knee pain decrease, VAS ≥ 20% analgesia), and the others as nonresponders (Fig 2B). Knee OA pain and the OA-specific pain and disabilities score (WOMAC questionnaire outcome) remained unchanged for nonresponders, while responders showed a mean decrease of 54.3% (95% confidence interval [CI] 29.7–79.0) in knee VAS pain and a mean 38.6% (95% CI 18.0–59.2) decrease in WOMAC score (Fig 2C and 2F) (note that throughout the study we use VAS scores for classification and WOMAC as an unbiased alternative outcome measure for knee pain). In comparison to the matched study 3 no-treatment observational group (in which 4 of 20 would be classified as responders to no treatment, based on VAS ≥ 20% analgesia) (see S1 Fig and S2 Table), placebo responders showed a large decrease in knee VAS pain in 2 wk (Fig 2D). After a 2-wk washout period (withdrawal of placebo pills), only knee VAS pain was obtained via a follow-up phone call, and placebo responders showed reversal of analgesia (analysis of variance [ANOVA]: interaction group x time F_(2,30)_ = 23.37, *p* < 0.001).

### Brain Regional Degree Counts Predicting Placebo Analgesia

**Fig 3:**
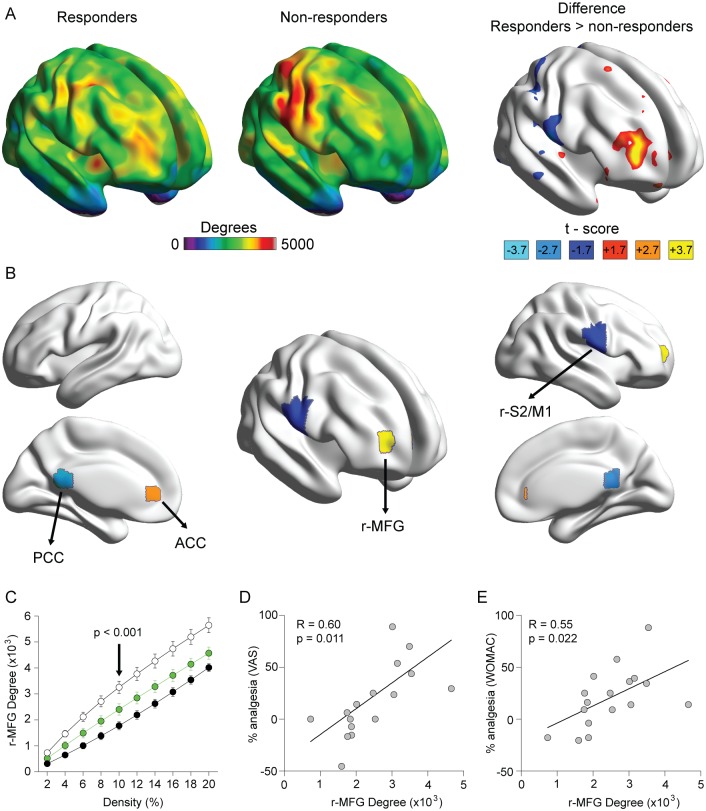
Patterns of brain connectivity in placebo responders and nonresponders in study 1. (A) Average brain maps for degree count (number of connections of any location to the rest of the brain), shown at 10% density in placebo responders (*n* = 8) and non-responders (*n* = 9), and the difference map. Placebo responders have higher (red to yellow colors) or lower (dark to light blue) degree counts than nonresponders. (B) The brain regions highlighted were identified based on minimal *t*-score and threshold-free cluster enhancement (TFCE) correction. The right midfrontal gyrus (r-MFG; x = 28, y = 52, z = 9 mm; cluster 12 voxels, *t*-score 3.7 or *p* < 0.001) was the region with the highest significant between-group difference, while bilateral anterior cingulate cortex (ACC; x = −3, y = 40, z = 2; cluster 10, *t*-scores 2.7 or *p* < 0.01), posterior cingulate cortex (PCC; x = −1, y = −45, z = 15; cluster 14, *t*-score −2.7 or *p* < 0.01), and a right region overlapping the secondary somatosensory and primary motor cortex (r-S2/M1; x = 60, y = −7, z = 21; cluster 31, *t*-score of 1.7 or *p* < 0.05) had lower significant differences. (C) Degree counts derived from r-MFG region in OA patients classified as placebo responders and nonresponders, and in healthy subjects (*n* = 20), for densities 2%–20%. At all densities, placebo responders (white) show higher degree counts than placebo nonresponders (black, group * density F_9,135_ = 15.3, *p* < 0.0001) or healthy controls (green, F_9,234_ = 5.8, *p* < 0.0001). (D, E) r-MFG degree counts at 10% density significantly predicted future (2-wk) magnitude of % analgesia for all OA patients based on both VAS and WOMAC scores. In C–E; black, gray, and white symbols represent the same groups as in Fig 1, while green symbols represent the healthy controls on which brain imaging was performed.

In study 1, whole-brain degree count maps, collected before the start of treatment, were used to identify potential brain regional markers for placebo propensity. Group differences in whole-brain degree count maps between placebo responders and nonresponders identified four brain regions that differentiated placebo responders from nonresponders. The highest significant difference was seen for the right midfrontal gyrus (r-MFG) (*p* < 0.001), (Fig 3A and 3B). Degree counts derived from r-MFG showed higher connectivity to the rest of the brain for responders across all densities, with most significant difference seen at 10% density (Fig 3C). At this density, average per voxel degree count within r-MFG was twice as high in responders as in nonresponders (3,256 ± 237 SE versus 1,777 ± 157 SE; t_15_ = 5.3, *p* < 0.001). Similar density-dependent degree count group differences were also seen for the other three brain regions (posterior cingulate cortex [PCC], anterior cingulate cortex [ACC], and the right secondary somatosensory and primary motor cortex [r-S2/M1]). The r-MFG counts explained placebo analgesia magnitude for all participants, based on VAS and on WOMAC changes from baseline (Fig 3D and 3E). Furthermore, to diminish the possibility that the r-MFG counts are related to a regression to the mean phenomenon (rather than a placebo pill response), we examined whether the r-MFG counts reflected symptom severity at time of entry into the study. We found that r-MFG counts were not correlated with VAS prior to treatment but became correlated with placebo treatment, and a similar pattern was also observed for WOMAC (S3 Table). Therefore, the r-MFG fulfills all the requirements for potentially predicting clinical placebo response, which we sought to validate in study 2.

### Placebo and Active Drug Analgesia in a Randomized Trial (Study 2)

Even though results in study 1 suggest that clinical placebo response is predictable and reversible, the results are based on a relatively artificial setting and a single-blinded study, designed to explore predictability of clinical placebo response. Thus, it was deemed necessary to test whether these findings can be generalized to the more natural setting of the standard clinical double-blind placebo versus active drug comparison scenario. To this end, we performed study 2, in which the primary objective was to test-validate the results obtained in study 1.

**Fig 4:**
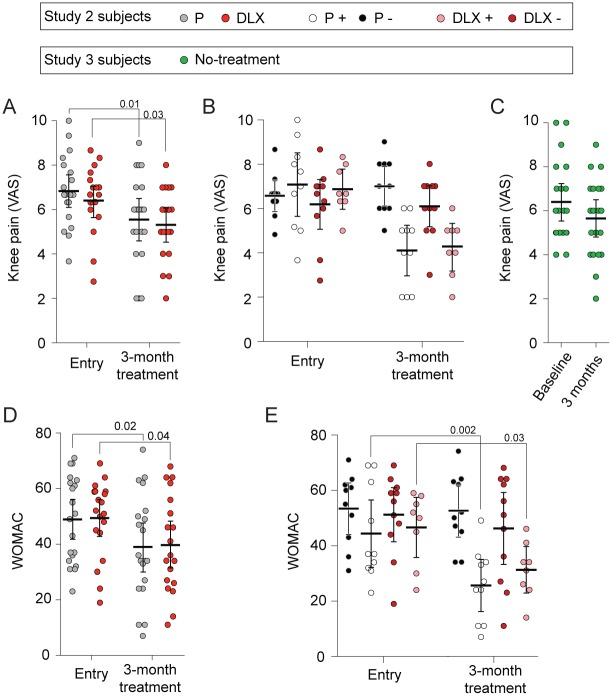
Pain relief in the double-blind placebo-controlled 3-mo duloxetine treatment, study 2. (A) Of all the knee OA pain patients who participated in study 2, 20 were randomized to placebo (P, grey) and 19 to duloxetine (DLX, red) treatment. Both groups started at the same level of knee pain (VAS) and exhibited significant and similar magnitudes of pain relief with a 3-mo treatment (only time-effect repeated-measures ANOVA, F_1,37_ = 14.8, *p* < 0.0001). (B) Participants in both arms were classified as responders (P +, white; DLX +, pink) or nonresponders (P −, black; DLX −, red) (≥20% analgesia over the 3-mo placebo treatment). Both treatments resulted in similar numbers of improvers and similar magnitudes of pain relief, observed, by design, only in the treatment responders (white and pink). (C) Twenty knee OA patients (study 3), matched for age, gender, and knee VAS pain at baseline, followed over 3 mo with no treatment (green). There was no within-group change in knee pain over 3 mo of no treatment. (D, E) We observe the same pattern of symptom relief, as observed for VAS, when the WOMAC scale is used as an outcome measure (only time-effect repeated-measures ANOVA, F_1,37_ = 13.3, *p* = 0.001). Error bars are 95% CIs. The illustrated *p*-values are post hoc comparisons that were statistically significant.

In study 2, the extent of pain relief, as measured by VAS or WOMAC (Fig 4A and 4D), was similar for placebo and duloxetine treatments. Also, the number of treatment responders (based on individual knee pain decrease over the course of a 3-mo treatment, VAS score ≥ 20% analgesia) did not differ between patients randomized to placebo (10/20, 50%) and patients randomized to duloxetine (8/19, 42%) (Fisher’s exact test *p* > 0.75). The magnitude of pain relief in treatment responders also did not differ between treatment groups (Fig 4B and 4E) on both VAS and WOMAC outcome scores. Importantly, in comparison to the matched study 3 no-treatment observational group (in which 7 of 20 would be classified as responders to no treatment at 3 mo, based on VAS ≥ 20% analgesia) (Fig 4C, S1 Fig, and S2 Table), placebo and duloxetine groups showed a larger decrease in the magnitude of knee VAS pain in 3 mo. Thus, the subjective self-report pain-related outcomes did not differentiate between placebo and duloxetine treatments but did show better analgesia for placebo and duloxetine treatment in contrast to no treatment.

### Validation of Placebo Predictability with Brain Functional Connectivity across Clinical Trials

**Fig 5:**
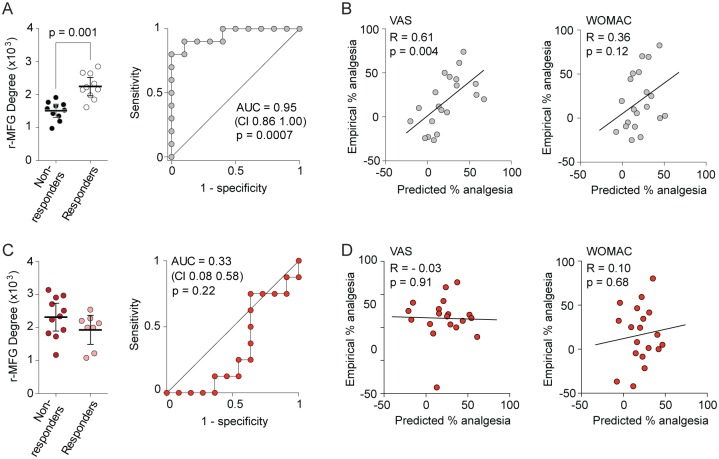
Predicting placebo and duloxetine treatment outcomes from r-MFG degree counts in study 2. Prediction of future outcomes (A, B for placebo treatment; C, D for duloxetine treatment) was assessed for r-MFG degree counts (based on brain coordinates derived from study 1). (A) r-MFG degree counts were significantly higher in placebo responders (post hoc honestly significant difference [HSD] test, *p* = 0.001), and the receiver operating characteristic (ROC) curve identified grouping at 95% accuracy. (B) Empirical analgesia was correlated to analgesia predicted from the best-fit line calculated in study 1, using r-MFG degree counts from study 2, for VAS (*p* = 0.004) and more weakly for WOMAC (*p* = 0.12) outcomes. (C) In contrast, in patients randomized to duloxetine, the r-MFG degree count did not differentiate between responders and nonresponders (t-score_17_ = 1.5, *p* = 0.17; ROC area under the curve [AUC] = 0.67) and (D) did not predict empirical analgesia for VAS and WOMAC outcomes. Error bars are 95% CIs. Symbol colors represent the same groups as in Fig 3.

To establish the generalizability of the primary brain marker for placebo response in study 1, we tested whether placebo predictive properties of r-MFG could be captured in study 2 participants. To ensure that the measure remained unbiased, only r-MFG degree counts were extracted for all study 2 subjects, obtained from the functional rs-fMRI scans performed before any treatments were dispensed. In study 2 OA patients who received placebo treatment, r-MFG degree counts were significantly higher in placebo responders (t_18_ = 4.9; *p* = 0.0001) (Fig 5A, left) and differentiated between placebo responders and nonresponders at 95% accuracy (Fig 5A, right). Perhaps more importantly, empirical placebo analgesia could be predicted from r-MFG degree counts when the best-fit linear regression equations, identified in study 1 between r-MFG degree counts and future placebo analgesia, were applied to study 2 r-MFG degree counts, both for VAS (*p* = 0.004) and WOMAC (*p* = 0.12) scores (Fig 5B, left and right). Therefore, the placebo response predictive properties of r-MFG degree counts were validated for the placebo treatment arm of study 2. Once again, to discount that r-MFG counts are a reflection of regression to the mean, we correlated the r-MFG values from study 2 placebo group with VAS and WOMAC values before treatment start and 3 mo after treatment. For both outcome measures, r-MFG counts were only correlated to knee pain after a 3-mo exposure to placebo pill treatment (S3 Table). We additionally explored, in multiple regression models, the contribution of demographics (age, gender, and pain duration), Beck Depression Inventory (BDI), Pain Catastrophizing Scale (PCS), past use of medications, and the r-MFG degree counts to explain VAS-based analgesia. The r-MFG degree count was the main contributor, explaining 37.5% of the variance. Age and gender also significantly contributed to the model by explaining 18.8% and 9.7% of unique variance, respectively. Performing the same analysis for WOMAC-based analgesia did not identify any additional contributions besides the r-MFG degree counts to the analgesia outcome.

### Modeling Predicted Placebo in Subjects Receiving Active Drug

Given that in study 2 there was no difference in pain outcomes—VAS or WOMAC—between placebo and duloxetine treatments, one would conclude that duloxetine is no better than placebo for pain relief, at least in the OA patients in study 2. The conclusion in turn leads to the hypothesis that r-MFG degree counts in the duloxetine arm, when entered into the regression equations derived from study 1, should just as accurately predict analgesic outcome as in the placebo treatment arm in study 2. In fact, this hypothesis was proven false. Degree counts in r-MFG did not differentiate between duloxetine responders and nonresponders. A two-way ANOVA for r-MFG degree count as a function of treatment type (placebo or duloxetine) and response type (responders and nonresponders) indicated a significant interaction (two-way ANOVA, F_1,34_ = 12.60, *p* = 0.0012); a post hoc Tukey HSD test indicated response type was significant for placebo treatment (HSD test = 5.18 with Studentized Range critical *p* = 0.001 threshold of 5.09), but not for duloxetine treatment (HSD test = 2.01 with 0.05 threshold of 2.87) (Fig 5C, left panel). Comparison of the receiver operating characteristic (ROC) curves obtained for placebo treatment (Fig 5A, right panel) and for duloxetine treatment (Fig 5C, right panel) indicated that they are significantly different (difference of 0.62, 95% CI (0.578–0.662), *p* < 0.0001), and applying the regression equation from study 1 to r-MFG degree counts in study 2 did not predict empirical analgesia for duloxetine treatment, both for VAS and WOMAC scores (Fig 5D). This result demonstrates that the duloxetine treatment and placebo treatment outcomes are differentiable at the brain circuitry level, although clinically they may be indistinguishable.

The bedrock assumption of all randomized controlled clinical trials is that placebo and active treatment responses are linearly additive [27] (more complex interactions may also exist, for example [28]), that is,

Empirical analgesia = Placebo response + Drug response.

This model is inherently assumed in all clinical trials, as the primary statistical test in randomized clinical trials is always a competition between the effect sizes of the two responses. The dissociation between our reported pain relief and r-MFG degree counts in the placebo- and duloxetine-treated subjects suggests that this linear additive relationship may not always be valid. We therefore pose the model as a formal hypothesis and examine its implications regarding (1) observed versus expected analgesia and (2) underlying brain information sharing properties.

### Relating Observed and Predicted Analgesia for Duloxetine Treatment

Given that the placebo arm of study 2 was fully explainable from study 1 results, r-MFG degree counts in the duloxetine treatment arm must also reflect the magnitude of placebo response in the patients randomized to active treatment. Thus, one can calculate a predicted placebo response in the duloxetine arm using the regression equation derived from study 1 and based on r-MFG degree counts in the duloxetine-treated patients. The above equation then becomes the following:

Empirical analgesia=Predicted placebo response + Drug response + Error.

**Fig 6:**
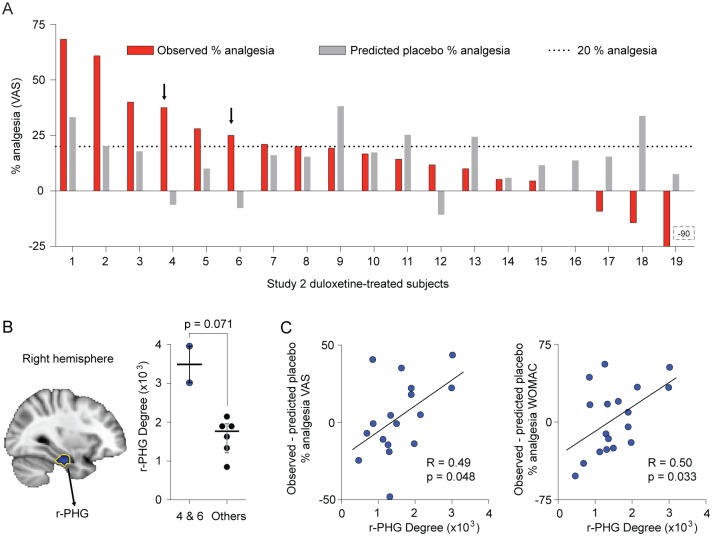
Right parahippocampal gyrus (r-PHG) degree counts predict future duloxetine response, based on modeling the placebo response in duloxetine-treated patients in study 2. (A) The empirical analgesia of individual duloxetine-treated patients (red) and the predicted placebo response (grey) are illustrated. The predicted placebo response was derived from the best-fit equation from study 1, which was applied to r-MFG degree count in duloxetine-treated patients. Patients with minimal predicted placebo and ≥20% empirical analgesia were considered mostly duloxetine responders (subjects 4 and 6; arrows). (B) Contrasting the whole-brain degree counts of these two subjects with the six other duloxetine responders (subjects 1, 2, 3, 5, 7, and 8) revealed a right parahippocampal gyrus region (r-PHG) in which degree counts were higher in subjects 4 and 6 (scatter of individual values and median and quartiles are shown; Mann-Whitney U-test, *p* = 0.071). (C) r-PHG degree count correlated with the difference between empirical analgesia and predicted placebo response for VAS (*p* = 0.048) and WOMAC (*p* = 0.033) outcomes, suggesting that the regional functional connections also reflect future placebo-corrected drug response for all 20 duloxetine-treated patients.

The Error term would have contributions from all parameters of the equation, at least because of measurement errors, and remains unknown. On the other hand, the individual subject predicted placebo response compared to the empirical analgesia provides an estimate of individual participant drug response (assuming that Error = 0 in above equation) (Fig 6A). We observe in Fig 6A that relative to the individual predicted placebo analgesia (gray bars), ingestion of duloxetine appears to have increased observed analgesia in subjects 1–6 and had no visible additional effect in subjects 7, 8, 10, and 14–16, while in subjects 9, 11, 13, and 17–19, duloxetine actually diminished the modeled placebo analgesia. Our model thus unravels the extent of efficacy of the active drug after correcting for modeled placebo responses. Moreover, these results indicate that a purely additive model cannot hold for the current data because only in one subgroup did duloxetine treatment increase observed analgesia from predicted placebo analgesia, while in another subgroup, it interfered with and diminished expected placebo analgesia.

### Identification of a Brain Region Predictive of Placebo-Corrected Drug Response

Banking on the notion that r-MFG is reflecting predicted placebo response, in the duloxetine-treated subjects we can estimate the expected duloxetine-related analgesia from the difference between predicted placebo response and observed analgesia (assuming Error = 0 in the linear equation). The difference between the red and grey bars in Fig 6A can then be considered the active drug treatment-related estimated analgesia after correcting for modeled placebo analgesia. Therefore, this difference provides the metric with which we test for the existence of a brain region predictive of future placebo-corrected response to duloxetine. Note the fact that the failure to differentiate behaviorally between placebo and drug response has no direct bearing on the hypothesis that a drug response prediction can be constructed by linearly modeling away the expected placebo response. Both duloxetine and placebo responses are mediated through central mechanisms; as a result, the interaction between and across identifiable brain regions may in turn explain their pain relief relationships.

In two participants (4 and 6), there was minimal predicted placebo response but above threshold empirical analgesia (20% analgesia dotted line), suggesting that these subjects were the drug responders with the least contribution from modeled placebo response. Therefore, a brain area with higher degree counts for these two subjects compared to the rest of the duloxetine responders (subjects 1–3, 5, and 7–8) may identify a brain region specific to drug treatment propensity. This search resulted in pinpointing the right parahippocampal gyrus (r-PHG) where the degree count was higher in subjects 4 and 6 from the remaining six duloxetine responders (Fig 6B). To assess the validity of this region, the r-PHG degree count was extracted for all duloxetine-treated subjects and correlated to the difference between empirical VAS analgesia and predicted placebo response (i.e., estimated placebo-corrected drug response). This correlation was significant (*p* = 0.048) for the duloxetine-treated group (Fig 6C left). When the same analysis was done for the unbiased WOMAC outcome measure (based on the best-fit equation derived from study 1 for WOMAC), it too showed a significant correlation between r-PHG degree counts and the difference between empirical WOMAC analgesia and predicted placebo response (*p* = 0.033) (Fig 6C right). Both correlations reinforce the idea that the r-PHG degree counts reflect/predict future drug responses, after accounting for the modeled placebo response.

In an exploratory multiple regression analysis, we examined the contribution of demographics, BDI, PCS, and past use of medications on the relationship between r-PHG and the difference between empirical analgesia and predicted placebo response, using VAS or WOMAC measures; no additional significant contributions were identified.

## Discussion

In OA patients in the setting of a randomized clinical trial and without manipulating expectations, we demonstrate that baseline brain connectivity can predict placebo responders and placebo analgesia magnitude. The approach leads to uncovering a potential detrimental effect of active treatment on expected placebo response and identification of brain connectivity predicting a drug response corrected for expected placebo response. The methodological approach advanced should be applicable to clinical trials in general and to the study of various types of placebo and treatments and suggests potential utility in clinical decision making.

The extent of information sharing (degree count) between a prefrontal cortical region, r-MFG, and the rest of the brain (obtained before start of treatment) best reflected the placebo response. We identified r-MFG from a 2-wk single-blinded clinical trial, in which the placebo analgesia was larger than in the no-treatment observational group and in which the placebo response was reversed after withdrawing placebo pills, and prospectively generalized its placebo predictive properties to a double-blind 3-mo treatment clinical trial. The r-MFG counts were not related to disease burden prior to start of placebo treatment in studies 1 and 2, diminishing the possibility that the measure is related to regression to the mean rather than a true placebo response. Therefore, r-MFG connectivity is likely a neural marker for placebo pill response in the clinical trial setting in OA. The robust validation of placebo response (95% accuracy) in the placebo-treated arm of study 2 enabled us to model away the expected placebo response in the drug treatment arm of study 2. This approach demonstrated the differential variability in drug- and placebo-induced analgesia. Moreover, as a proof of concept, we separated drug responders from mixed—i.e., drug and placebo—responders, and this then led to uncovering a brain region, the r-PHG, with properties consistent with being predictive of future drug response presumably uncontaminated with placebo response (r-PHG was identified using a contrast of two versus six participants, and its properties could then be generalized to the whole group of 19 patients that received duloxetine treatment). To our knowledge, this is the first study identifying a brain site that appears to predict future placebo-corrected drug response propensity, even when the verbal reports did not distinguish between placebo and drug treatment effects. The r-PHG predicting placebo-corrected drug response should be considered an exploratory result, as it awaits future validation (especially since in the linear model we assumed the error term to be zero, and the linear model was an assumption that remains untested relative to more complex models). On the other hand, the neurobiological properties of r-PHG that dictate its functional connectivity to the rest of the brain should match the pharmacology of the action of duloxetine in the brain, and the observation suggests the general concept of the possibility of matching patients with treatments given their brain propensities across therapeutic options.

There is a substantial body of literature regarding mechanisms of placebo response [6,7,11,12,29–31]. However, studies regarding predicting placebo response in the clinical trial setting and for chronic pain patient populations are sparse [21,32,33]. The present study is a major departure from the standard approach in which placebo is assessed for acute painful stimuli while expectations are manipulated [9,13,34–36]. Placebo effects observed in the clinical settings in the past have been ascribed to statistical rather than biological factors [8,37,38]. Putative explanations include (1) improvement being due to the natural history of the disease, (2) improvements reflecting regression to the mean, and (3) patients benefiting from the positive psychosocial context of being enrolled in a study. We captured some of such influences in study 3 and observe over time a smaller nonspecific OA knee pain relief than during placebo treatments. Consequently, our study results suggest that specific brain biology underlies the clinical placebo effect. Leading investigators in the field of placebo research have repeatedly wondered whether placebo response is predictable. As Benedetti [37] states, “A central issue in placebo research is whether an individual in whom placebo works possesses one or more specific characteristics, which can reliably identify him *a priori* as a placebo responder.” Similarly, another author poses the question: “Do placebo responders exist?” [39]. Here, we show results consistent with the notion that in OA subjects, the placebo pill responder is predictable, and her/his brain connectivity determines a priori both propensity and magnitude of improvement.

Clinical placebo analgesia is only studied in a few neuroimaging studies in back pain, fibromyalgia, and OA [13,21,23]. Diversity of patient types and analysis methods obviate direct comparison of outcomes, yet in all cases, prefrontal circuitry is implicated. The current study is the first to employ a rigorous whole-brain contrast regarding brain network information sharing in placebo responders and identifying and validating a biomarker predictive of placebo response. To uncover perceptual correlates to this placebo response brain marker, we relied on a meta-analytic approach [40]. Based on 525 studies, the reverse-inference highest-probability terms associated with r-MFG activity were observed to be related to decision making, memory, and planning. These associations support the idea that placebo analgesia is driven by top-down modulation [22], presumably integrating prior experience to define future placebo responses. Given our limited dataset, we intentionally constrained the analysis to the most robust predictor of placebo response. However, three additional brain regions were also predictive of placebo response in study 1. Increased connectivity of the ACC and decreased connectivity of the PCC in placebo responders suggest a shift of balance in attentional circuitry, while decreased connectivity of the sensorimotor cortex implicates sensory, perhaps nociceptive, processing and motor behavior modulation in placebo propensity. The complexity of the identified circuitry is consistent with long-standing evidence that placebo effects underlie a multiplicity of mechanisms [7] and that the identified circuitry may be specific to the condition (OA knee pain) and to the specific placebo response studied (ingestion of pills). The reverse-inference highest-probability terms associated with the brain region predicting future drug response—i.e., r-PHG—were related to memory encoding and retrieval, as well as to sadness and negative emotions. Whether these memory and emotion-related characteristics could predict duloxetine responsiveness remains to be tested.

The linear placebo response model we employed uncovered variability in placebo-corrected duloxetine responses. Observed responses can be grouped into three categories: (1) the drug improving placebo response (6/19), (2) the drug having no obvious additional effect beyond that of the placebo (7/19), and (3) the drug diminishing expected placebo responses (6/19). Similar to almost all centrally active drugs, duloxetine is associated with a long list of side effects. Here, we uncover a hitherto unobservable side effect, namely, the active drug diminishing the placebo response. The specific mechanism underlying duloxetine interference on placebo response remains to be studied; yet, it might be mediated by engaging r-PHG, which in turn either disturbs connectivity of r-MFG (and/or other placebo predictive regions) or modulates targets critical to the r-MFG connectivity.

### Conclusions, Limitations, and Future Directions

One weakness of the current study is the limited number of subjects used, counterbalanced by the reversibility of placebo response during washout and a robust validation and by showing the superiority of placebo analgesia relative to the no-treatment group both at 2 wk and at 3 mo, which altogether distinguish clinical pill placebo in OA from statistical confounds. An important design limitation of our study was the independent recruitment for study 3. Ideally, the no-treatment-arm patient entry should have been randomized within study 1 and study 2 recruitments. We should also qualify that observed analgesia seen in placebo responders may still be considered (although unlikely) a reflection of natural recovery or symptom fluctuation rather than definitely caused by the placebo, because study 3 subjects were not randomized into study 1 and 2, pain ratings were only collected at entry and end of treatment, and the washout period knee pain was collected over the phone (thus, the rapid and complete reversal of analgesia may be due not just to cessation of pill administration but also to the change in environmental cues). Even though we used a single threshold (20%) for defining placebo responders, the identified brain property—degree counts of r-MFG—identifying placebo responders could also predict the continuous measure of magnitude of placebo analgesia for VAS and for WOMAC in study 1 and study 2, implying that the identified brain marker is not strictly dependent on the specific analgesia threshold chosen. The ubiquity or specificity of the brain marker uncovered for placebo response in OA remains to be identified across types of chronic pain and for various placebo-type manipulations. Moreover, the predictability of future drug response after modeling away the placebo response requires replication and trial designs in which the error term in the linear equation can be systematically estimated and the general applicability of the linear model tested in contrast to more complex model designs (e.g., equations incorporating multiplicative or higher order polynomial terms), as well as testing for drug-type specificity.

The current study falls within the general effort of using neuroimaging technology to forecast the future health status of individuals, which is showing predictive value across many medical domains [18]. The opportunity presented with identification of placebo and placebo-corrected drug response predictive brain markers, specifically in chronic pain patients and for clinical trials using neutral instructions, presents both a concrete and humanitarian possibility of decreasing suffering with the recognition and identification of individual differences in brain function. If future similar studies can further expand and eventually provide a brain-based predictive best-therapy option for individual patients, it would dramatically decrease unnecessary exposure of patients to ineffective therapies and decrease the duration and magnitude of pain suffering. Moreover, if placebo response can be predictably removed/reduced in clinical trials, then, besides reducing the cost of clinical trials, the efficacy and neurobiology of therapies can be identified more accurately and at the level of the individual.

## Methods

### Participants

All participants gave written informed consent to procedures approved by the Northwestern University Institutional Review Board committee (STU00039556 for study 1 and 2, STU00059872 for study 3). We recruited a convenience sample of 143 community-based people with knee osteoarthritis (OA) through public advertisement and Northwestern University-affiliated clinics. A total of 20 patients were recruited for study 1, 70 for study 2, and 53 for study 3. From these, 45 either did not complete the studies they were enrolled in or their brain scans did not pass the quality assessment pipeline (see Fig 1, S1 and S2 Tables for demographics). Of the remaining 98 patients, 17 took part in study 1, 39 in study 2, and 42 in study 3 (from which 20 were selected to match our other groups). We also recruited 20 age-matched healthy control subjects. Healthy subjects were matched to the mean age and gender distribution of all OA patients. All OA participants met the American College of Rheumatology criteria for OA (confirmed by TJS) and had pain of at least 1-y duration. A list of inclusion and exclusion (mainly presence of other chronic pain conditions and major depression) criteria was imposed, including a knee-pain intensity of at least 4/10 on the 11-point numerical rating scale (NRS) within 48 h of the screening visit. A detailed list of all inclusion and exclusion criteria as well as clinical trial registrations is presented in the Supporting Information file (see S1 Text).

### Study Design

Data from three different studies were used in the manuscript (Fig 1). Study 1 (Clinicaltrials.gov accession number: NCT02903238; protocol details in S1 Study Protocol; relevant checklists in S1 and S2 Checklists) constituted the discovery group and was used to identify and localize brain functional differences between placebo responders and nonresponders. All study 1 participants ingested placebo pills (lactose) once a day for 2 wk in a single-blind design. Prior to the experiment, participants were informed that they would have an equal chance of receiving a placebo pill or an active drug. The research staff knew that all patients were receiving placebo pills. Pain and behavioral parameters were collected in person before and after treatment, and knee pain VAS scores were additionally collected 2 wk after drug washout via a phone call. For all patients, brain scans were collected prior to treatment.

Study 2 (Clinicaltrials.gov accession number: NCT01558700; protocol details in S1 Study Protocol; relevant checklists in S1 and S2 Checklists) was performed independently and after the end of study 1. This study served as the validation group and involved a double-blinded trial in which patients received placebo or duloxetine for 3 mo. Study 2 participants ingested either placebo pills or duloxetine at a dose of 30 mg for the first week and escalated to 60 mg for the rest of the treatment period, except for the last week, when the dose was decreased back to 30 mg. Drug preparation was made by an independent clinical research assistant, and the research staff providing treatment to patients were kept blind at all times about subjects’ treatment. For this study, a parallel assignment intervention model was used, with a simple randomization using a 1:1 allocation ratio. Randomization codes were prepared by an independent clinical research assistant under TJS’s supervision, used for drug preparation, and then concealed until the end of the study. The research staff performing recruitment and collecting data were never in contact with the randomization codes until the end of the study. The main purpose of study 2 was to validate the placebo propensity marker found in study 1, but in a real clinical trial environment to mimic what is normally performed (e.g., by pharmaceutical companies) for drug efficacy assessment. Another aim of study 2 was to test the specificity of the brain biomarker to treatment type (i.e., whether the biomarker identified in study 1 reflects general response to treatment or placebo response specifically). Behavioral and clinical parameters were obtained before and after treatment. Brain scans were collected prior to treatment. For study 1 and 2, patients were asked to discontinue their medications 2 wk prior to the beginning of the trial and were provided with acetaminophen as rescue medication. In this study, two duloxetine-treated and three placebo-treated patients reported worsening of knee pain; four duloxetine-treated and three placebo-treated patients reported dizziness and grogginess symptoms. No serious adverse events were reported.

Study 3 (protocol details in S2 Study Protocol) was also run independently from study 1 and 2. For this study, participants did not receive any medications and were asked to continue their regular treatment regimen. Patients from study 3 represent the natural progression of the OA condition within the same time frame as study 1 and 2. The purpose of study 3 was therefore to account for regression to the mean or any other biases that could influence the outcome of the subjects’ report of OA pain in the clinical trial setting.

### Behavioral and Clinical Measures

Patients from all studies completed a general health questionnaire and a VAS (on a 0 to 10 scale) for their knee OA pain. Patients from study 1 and 2 also completed the WOMAC, the BDI, and the PCS. All questionnaires were administered on the day of brain scanning. Response categorization and brain regions of interest were identified using only the VAS measure and then tested for consistency using WOMAC. Thus, WOMAC provided an unbiased estimate of treatment response and of brain regional properties. Analgesic response was defined a priori on an individual basis as at least a 20% decrease in VAS pain from baseline to the end of treatment period; otherwise, subjects were classified as nonresponders. This threshold for analgesic response was chosen based on our earlier results [15] and also based on a recent meta-analysis estimate of the size of placebo analgesia [32]. In study 2, to partially compensate for regression to the mean effects, VAS was measured 3 times over a 2-wk period prior to the start of treatment and after cessation of medication use, averaged, and used as the indicator of pain at entry (designated as baseline in the figures).

### Brain Scanning Parameters (Studies 1 and 2)

For all participants in studies 1 and 2, MPRAGE type T1-anatomical brain images were acquired as described before [15]. Briefly, a 3T Siemens Trio whole-body scanner with echo-planar imaging (EPI) capability using the standard radio-frequency head coil with the following parameters: voxel size 1 × 1 × 1 mm; TR = 2,500 ms; TE = 3.36 ms; flip angle = 9°; in-plane matrix resolution = 256 × 256; slices = 160; and field of view = 256 mm. rs-fMRI images were acquired on the same day and scanner with the following parameters: multi-slice T2*-weighted echo-planar images with repetition time TR = 2.5 s, echo time TE = 30 ms, flip angle = 90°, number of slices = 40, slice thickness = 3 mm, and in-plane resolution = 64 × 64; the number of volumes was 300. The 40 slices covered the whole brain from the cerebellum to the vertex. All MRI data are available on openfmri.org.

### fMRI Preprocessing and Data Analysis (Studies 1 and 2)

As we described before [15], the preprocessing of each subject's time series of fMRI volumes was performed using the FMRIB Expert Analysis Tool (FEAT [41], www.fmrib.ox.ac.uk/fsl) and encompassed the following: discarding the first five volumes to allow for magnetic field stabilization, skull extraction using BET, slice time correction, motion correction, spatial smoothing using a Gaussian kernel of FWHM 5 mm, and high-pass temporal filtering (150 s). Several sources of noise, which may contribute to non-neuronal fluctuations, were removed from the data through linear regression. These included the six parameters obtained by rigid body correction of head motion, the global BOLD signal averaged over all voxels of the brain, signal from a ventricular region of interest, and signal from a region centered in the white matter.

All preprocessed fMRI data were registered into standard MNI space multiplied by a common gray matter mask generated from all subjects in the study (this step was performed in order to limit all analyses to a common set of gray matter voxels) and subsequently down-sampled to yield 29,015 regional cortical and subcortical nodes (4 x 4 x 4 mm isometric voxels).

### Brain Graph Construction (Studies 1 and 2)

To construct the whole-brain voxel-wise connectivity networks for each subject, we first computed the Pearson correlation coefficient (R) for all possible pairs of the 29,015 cortical and subcortical voxel time series from the preprocessed rs-fMRI data. For each subject, the threshold was calculated to produce a fixed number of edges M to be able to compare the extracted graphs [42]. Therefore, the values of the threshold are subject dependent. Each of these extracted graphs comprised N = 29,015 nodes corresponding to the number of voxels and M undirected edges corresponding to the significant nonzero absolute values of correlation greater than the value of the threshold. Since the value of the chosen threshold is important [42,43], we chose to test several values of threshold, from a conservative threshold corresponding to 2% connection density (the percentage of edges with respect to the maximum number of possible edges [(N x N– 1) / 2]) to a lenient threshold corresponding to 20% link density. Networks constructed at 2% link density are dubbed sparse networks, while those constructed at 20% are dense. In general, results are presented over a range of thresholds to give the reader a sense of the (lack of) dependence of a property upon thresholds, and no formal definition of threshold ranges is proposed since it is essentially arbitrary.

### Nodal Degree Difference between Responders and Nonresponders (Study 1)

To localize the nodes (voxels) that exhibited significant changes in number of connections (degrees) in responders and nonresponders in Study 1, we performed a whole-brain analysis. First, for each subject and link density, we computed the number of edges (links) for each node from the brain graph, using the BCT toolbox [44]. The number of degrees was used to construct single brain volume in standard MNI space for each subject, in which the value assigned for each node corresponds to the degree of that given node. Differences in nodal degree between responders and nonresponders, at each link density, were carried out using Randomise in FSL [45]. This technique uses permutation-based inference to allow for rigorous comparisons of significance within the framework of the general linear model with *p* < 0.05. Group differences were tested against 5,000 random permutations, using the threshold-free cluster enhancement (TFCE) method. The maps between responders and nonresponders were contrasted at all densities, and the final difference map represented the conjunction of significantly different voxels across at least eight out of the ten densities evaluated after TFCE correction. Finally, the conjunction map presented in Fig 2B was used to determine regions of interest that serve as brain biomarkers for placebo response.

### Validation of Brain Biomarkers (Study 2)

Pretreatment degree counts for the regions of interest identified in study 1 were determined for all study participants, and we tested whether these values can predict placebo analgesia and/or duloxetine analgesia in study 2. Prediction accuracy was tested using a binary (i.e., prediction of group responders versus nonresponders) or a continuous model (prediction of response magnitude). The significance of the binary classification was determined by an ROC area-under-the-curve (AUC) analysis that identifies the sensitivity and specificity of predicting future treatment outcomes. The significance of the continuous prediction was determined using a regression analysis of predicted versus observed outcomes. The relationship between outcome and degree was determined from linear fitting (y = ax + b) for study 1, where y = represents % analgesia response, a = fitted slope, and x = degree count of region of interest (ROI). This equation was used to determine the outcome (y2) in study 2 (y2 = ax2 + b), where x2 represents the degree count of the ROI. The significance of the prediction was assessed by correlation analysis between the predicted outcome (y2) and the observed response for patients in study 2.

### Additional Statistical Analysis

For Figs 2 and 4, a repeated-measures ANOVA compared between groups and time effects. Post hoc comparisons’ *p*-values (after Bonferroni correction for multiple comparison) are indicated on the figures. For Fig 5A and 5C, two-way ANOVA was used, and the post hoc *p*-value is indicated on the figure. For Fig 6B, a nonparametric test was used because the number of observations was small. Multiple regression analysis was performed with the linear regression tool in SPSS software. VAS analgesia was set as the dependent variable, and r-MFG count value, gender, age, pain duration, past medication use, and BDI and PCS scores were set as independent variables. The stepwise forward elimination method was then used to generate a multifactorial regression model, using a *p* < 0.05 criterion for adding variables and a *p* < 0.1 criterion for removing a variable and repeating the process until none improved the process. To compare between ROC curves in Fig 5, we used the online tool provided from varrstats (vassarstats.net/roc_comp.html), which is using the method described in [46]. This provided us with the difference and standard error. We then calculated the 95% CI using the following formula: CI = ± Z_α/2_ * σ / √(*n*), where Z_α/2_ represents the z-value at α/2 (where α is the confidence level, here 95%), σ is the standard error, and *n* is the sample size.

## Supporting Information

S1 Checklist Consolidated Standards of Reporting Trials (CONSORT) 2010 checklist of information to include when reporting a randomized trial following www.consort-statement.org requirements. (PDF)

S2 Checklist Transparent Reporting of Evaluations with Nonrandomized Designs (TREND) checklist of information to include when reporting a nonrandomized trial following www.cdc.gov/trendstatement/ requirements. (PDF)

S1 Data Raw data used in all figures. (XLSX)

S1 Fig Knee pain variation in the observational group, study 3, over time. Knee pain profile over time for the observational no-treatment group (study 3). To assess regression to the mean and other statistical effects on knee pain over time, an independent OA cohort (*n* = 53) was followed for a 3-mo period without any intervention. Participants’ knee pain (VAS) and related parameters were collected during four clinic visits. (A) From this cohort, 42 subjects completed the full trial. The pain ratings in these OA patients did not show a significant change over the 3-mo monitoring (one-way repeated-measures ANOVA, F(2.6, 106.1) = 2.6, *p* = 0.062). (B) A subgroup of 20 OA patients were additionally selected to match study 1 and study 2 age, gender, and mean baseline VAS pain. In this group also, there was no significant change in OA pain over 3 mo of monitoring (one-way repeated-measures ANOVA, F(2.4, 45.8) = 1.0, *p* = 0.38).
(TIF)

S1 Study Protocol Full Northwestern University protocol for study 1 and 2. (PDF)

S2 Study Protocol Full Northwestern University protocol for study 3. (PDF)

S1 Table Demographics for participants in study 1 and study 2 and healthy controls. Values are shown as mean and 1 SE (in parenthesis). Duration = duration of OA knee pain, in years; MQS, Medication Quantification Scale = medication use at time of entry into study.
(DOCX)

S2 Table Demographics and pain scores for participants in study 3. Values are shown as mean and 1 SE (in parenthesis). Duration = duration of OA knee pain, which was only available in 50% of participants; VAS = knee OA pain.
(DOCX)

S3 Table Relationship between knee pain and the brain placebo predictor, r-MFG degree counts, before and after placebo treatment. The degree counts from the r-MFG region extracted from scans for study 1 and study 2 (placebo-treated groups) were correlated with the knee pain values (VAS and WOMAC) obtained before and after the placebo treatment. No correlation was observed with VAS before treatment for both groups, while VAS after treatment significantly correlated with r-MFG degree count. A similar, but not as robust, pattern was observed for WOMAC as well. Data are shown as R-values (*p*-values are shown in parentheses, with significant correlations in bold). This analysis was performed to test the extent to which r-MFG counts may be reflecting regression to the mean. The obtained results are suggestive (but do not fully rule out) that r-MFG counts reflect the placebo response rather than regression to the mean.
(DOCX)

S1 Text Complete list of inclusion and exclusion criteria. (DOCX)

## Abbreviations

ACC: anterior cingulate cortex
ANOVA: analysis of variance
AUC: area under the curve
BDI: Beck Depression Inventory
BOLD: blood-oxygen-level dependent
CI: confidence interval
CONSORT: Consolidated Standards of Reporting Trials
EPI: echo-planar imaging
fMRI: functional magnetic resonance imaging
OA: osteoarthritis
PCC: posterior cingulate cortex
PCS: Pain Catastrophizing Scale
NRS: numerical rating scale
r-MFG: right midfrontal gyrus
ROC: receiver operating characteristic
ROI: region of interest
r-PHG: right parahippocampal gyrus
r-S2/M1: right secondary somatosensory and primary motor cortex
rs-fMRI: resting-state functional magnetic resonance imaging
TFCE: threshold-free cluster enhancement
TREND: Transparent Reporting of Evaluations with Nonrandomized Designs
VAS: visual analog scale
WOMAC: Western Ontario and McMaster Universities Osteoarthritis Index
