# Supplementary figures and images for: Brain Connectivity Predicts Placebo Response across Chronic Pain Clinical Trials
Source: PLoS Biol. 2016 Oct 27;14(10):e1002570. doi: 10.1371/journal.pbio.1002570 (PMC5082893; doi:10.1371/journal.pbio.1002570)

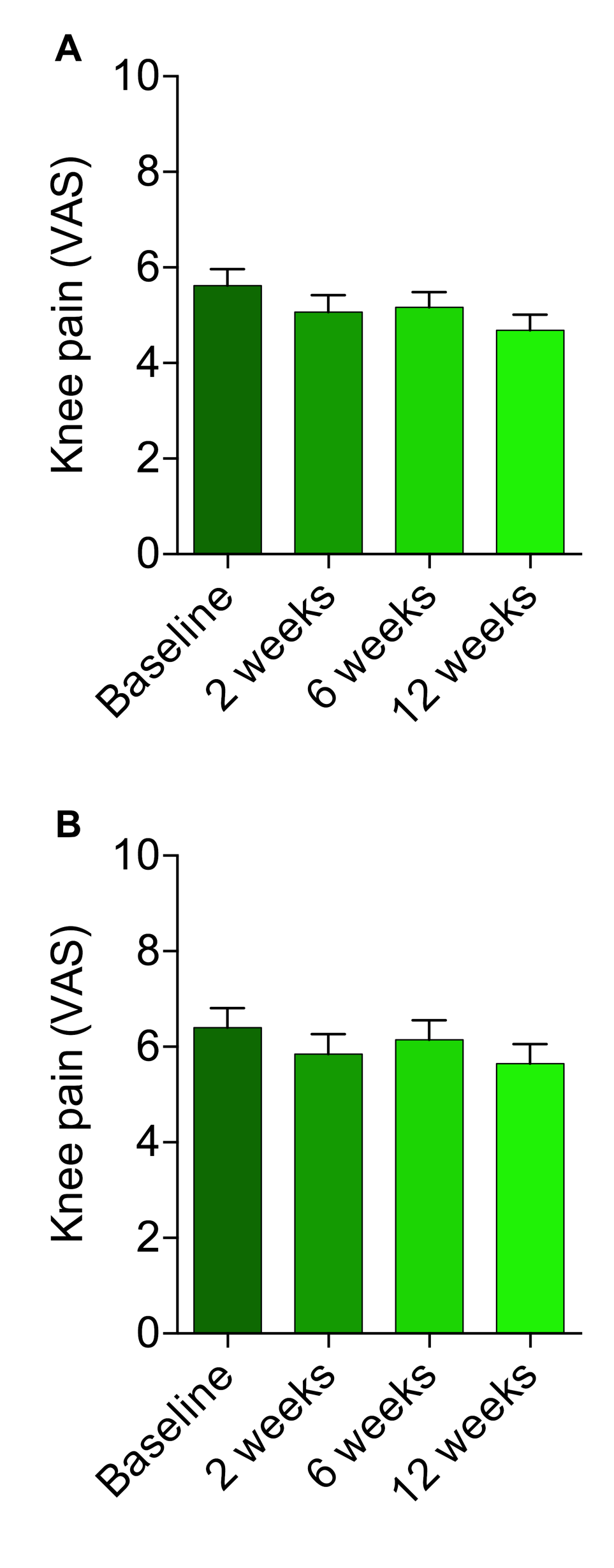

Supplement: S1 Fig — Knee pain profile over time for the observational no-treatment group (study 3). To assess regression to the mean and other statistical effects on knee pain over time, an independent OA cohort (n = 53) was followed for a 3-mo period without any intervention. Participants’ knee pain (VAS) and related parameters were collected during four clinic visits. (A) From this cohort, 42 subjects completed the full trial. The pain ratings in these OA patients did not show a significant change over the 3-mo monitoring (one-way repeated-measures ANOVA, F(2.6, 106.1) = 2.6, p = 0.062). (B) A subgroup of 20 OA patients were additionally selected to match study 1 and study 2 age, gender, and mean baseline VAS pain. In this group also, there was no significant change in OA pain over 3 mo of monitoring (one-way repeated-measures ANOVA, F(2.4, 45.8) = 1.0, p = 0.38). (TIF) [file pbio.1002570.s004.tif]
