# Supplementary material for: Brain Connectivity Predicts Placebo Response across Chronic Pain Clinical Trials
Source: PLoS Biol. 2016 Oct 27;14(10):e1002570. doi: 10.1371/journal.pbio.1002570 (PMC5082893; doi:10.1371/journal.pbio.1002570)
Supplement: S1 Study Protocol — (PDF) [file pbio.1002570.s005.pdf]

# Brain Morphometric Study in Knee Osteoarthritis Patients Treated with Duloxetine


## Principal Investigator: A. Vania Apkarian, PhD

**Address: 303 E. Chicago Ave, Physiology Dept. Ward Bldg, 7-705**
**Chicago, IL 60611**


## Sites of Study:

**Northwestern University**
**680 N. Lake Shore Drive**
**14th floor**
**Chicago, IL 60611**

**Northwestern Memorial Hospital**
**251 E. Huron**
**Chicago, IL 60611**

**Northwestern University**
**Olson Pavilion**
**710 N. Fairbanks Ct.**
**LC 0-225 (basement)**
**Chicago, IL 60611**

**Northern Ohio Neuroscience, LLC**
**5433 State Route 113, East**
**Bellevue, Ohio 44811**

## Background and Rationale:

This study and the hypotheses to be tested arise from work done in our group evaluating brain cortical changes in people with chronic back pain. These studies demonstrated a loss of about 1.5 cc of neocortical gray matter per every year of living with the condition (Apkarian et al.




NU IRB Approved
through «ExpireDate»

2004). This gray matter loss is in addition to the 2.5 cc of gray matter loss per year that is a consequence of normal aging. Since this original publication, more than ten studies have replicated the basic result and showed that distinct chronic pain conditions are associated with specific brain anatomical reorganization, characterized by brain regional decrease in grey matter density. Two recent studies have also shown that when the chronic pain is completely reversed the brain anatomical changes seem to at least partially reverse (Oberman et al. 2010, Raecke et al. 2009), within the time span of 4-12 months. The latter two studies provide evidence for a time window for brain anatomical reversal of grey matter abnormalities as well as an estimate of the number of subjects needed to detect such changes. However, they are based on populations where the pain condition is completely eliminated.

The latest advances suggest that this project is eminently possible and provide clear guidelines for its implementation. This proposal has not been submitted elsewhere and is not part of any other project.

A fundamental question that arises from these studies is the extent of reversibility of the brain atrophy associated with chronic pain following continuous use of an analgesic. Apkarian's lab has generated very strong evidence (submitted for publication) that the brain anatomy of subjects with OA is dramatically different from healthy subjects. Given that recent data show that joint replacement (in hip OA) reverses brain atrophy, we can now hypothesize with greater confidence that an effective analgesic should also reverse at least some of the brain atrophy observed in OA. Thus, a study in patients with chronic knee OA treated with duloxetine provides a unique opportunity to answer this question. Since in this study OA patients will have a single new agent for four months, one can directly examine the effects of treatment in relation to progression or regression of brain atrophy. Given the results of recent studies, four months should be an adequate time to study the effects of therapy on brain atrophy (we had originally suspected that one year of treatment was necessary to observe such changes). Moreover, since the subjects can be scanned at entry into the study and then at termination of study, this will provide a within subject contrast for changes in brain atrophy which reduces signal variability and provides the opportunity of relating extent of pain relief to changes in brain atrophy. When the pain is completely relieved, a group of 10 patients has been adequate to demonstrate reversal of brain atrophy (Oberman et al. 2010, Raecke et al. 2009).

The issue of atrophy due to pain in relation to changes due to aging warrants further clarification. In the 4 months of treatment all subjects should have atrophy due to aging, about 2.5 cc*0.3 = 0.75 cc. On the other hand the OA cumulative atrophy over the years seems to be proportional to the number of years a given subject has had the condition. If we assume on average that the mean OA duration is 5 years, then the atrophy due to chronic pain should be about 1.5 cc * 5 = 6.0 cc. Therefore, in a subject where the OA pain completely disappears with treatment, that subject has the potential of exhibiting a reduction in atrophy of 6.0 cc minus the increased atrophy due to aging of 0.75 cc. Moreover, we assume that placebo responses will not induce reversal of atrophy because placebo mainly reflects attentional and motivational states of the person. There is likelihood that this assumption may not be true. Even in the latter case as we expect placebo to decrease pain less than the real drug treatment, we should still observe smaller changes in atrophy by placebo.

We consider the brain atrophy in chronic pain as an overall marker of the extent of peripheral, spinal cord, and brain reorganization a subject has developed with living and suffering with the condition. Animal models of chronic neuropathic and inflammatory pain repeatedly show that peripheral and spinal cord nociceptive transmission is dramatically changed, leading to


**NU IRB Approved through «ExpireDate»**

peripheral and central sensitization. We and others have also shown recently in animals that a few days following peripheral nerve injury one observes morphological changes at the level of individual neurons, where number of spines and dendritic lengths increase together with increases in NMDA/AMPA ratio (Metz et al. 2009). We presume that these cortical changes are the same ones contributing to atrophy in human chronic pain. However, most of underlying mechanisms remain to be uncovered. Humans suffering from chronic pain exhibit a large number of cognitive and emotional deficits. We presume that these deficits are directly related to the brain atrophies discovered in chronic pain conditions. However, unfortunately there are no direct studies linking brain regional atrophies to cognitive abilities in chronic pain. Such preliminary studies are underway in Apkarian's lab but the results will not be available for quite a period. Thus, reversing this atrophy would correspond to reversing brain plasticity at multiple levels in the nervous system, and should also correspond to improvements in cognitive and emotional abilities.

## Hypotheses:

The primary hypothesis to be tested in the study is that subjects with chronic knee OA treated with Duloxetine over a four-month period will show decreased or arrested brain atrophy compared to subjects treated with placebo.

Secondary hypotheses are that in individual patients there will be a relationship between the magnitude of the analgesic effects of treatment and the magnitude of the change in brain atrophy. It is anticipated that there will be a correlation between pain relief by Duloxetine and changes in brain atrophy, whereas the degree of pain relief by placebo will not correlate with brain atrophy changes.

## Research Methods:

### *Study Design*

A placebo-controlled trial of **two parts** the first of which includes 20 patients with chronic knee OA that will be single blinded, and the second of which includes 60 patients (different from those included in the first part) with chronic knee OA that will be randomized in a 1:1 ratio to Duloxetine or placebo . The Second part (Phase II) will be started after the first part is completed (Phase I).

The total number of OA subjects, hence, culminates to a total of 80 patients. Subjects that terminate the trial before completion of the final visit and scan will be replaced. An additional cohort of normal subjects will also be studied in parallel during the second phase of the study, to allow evaluation of changes in brain volume over time.

In the first part of the study (phase I), all the participants will be receiving placebo. However this part (phase I) of the study will be single blinded. The participants will not know whether they are receiving placebo or active medication. They will be told that there is a possibility that they receive either medication. The researcher, however, will know that all participants in phase one are receiving placebo.


NU IRB Approved
through «ExpireDate»

In the second part of the study (phase II), 60 patients will be recruited and randomized into study drug/placebo in a 1:1 ratio in a double blinded manner where neither the subject nor the researcher will know whether the drug dispensed is active or placebo.

In the **first part** of the study, participants will undergo one high resolution anatomical scan (T1) and one functional scan (a resting scan) in a 3 Tesla magnet. A single scanning session comprised of a 10 minute functional scan (a resting scan) will be done two weeks from the first scan. After finishing this second scan (done two weeks after the initial scan), the participants in part one of the study will have completed their role in the study and will not be asked to show up for any other scans or follow-ups.

For the **second part** (Phase II) of the study, participants entering this part of the study will undergo one initial high-resolution anatomic scan (T1) and one functional scan (a resting scan) in a 3 Tesla magnet. A similar scanning session is then performed at the end of the study (approximately 4 months later).

For all of these scans, the participant only has to lie quietly and remain still in the scanner. Each scanning session (consisting of a total of two 10 minute scans) takes a little under 25 minutes to complete and is considered completely non-invasive. The only discomfort is the requirement that subjects lay still in the scanner for the duration. All patient participants complete a general health questionnaire (PHH), the short form of McGill Pain Questionnaire (sf-MPQ), the Western Ontario and McMaster Osteoarthritis Index (WOMAC), and the Beck Depression Index (BDI) at screening, the time of entry (baseline visit), and at the final scanning visit (visit 5). Only the WOMAC will be completed on interim visits three and four.

Healthy control participants will participate in part two of the study only. They will be asked to complete the PHH only at the screening visit (to make sure that there are no potential physical or mental comorbidities or confounding medications). They will be asked to personally complete the BDI at all visits, and they will have the same number and types of scans as OA participants.

All participants will also have the choice of completing an optional visit at the very end of the study that involves answering questions in an open-ended interview and completing a set of new questionnaires that supplement this interview. OA participants will be asked a series of questions about their chronic pain, mood, medical history, and medical beliefs which, depending upon how detailed their responses are, will last 30 minutes to an hour. After the interview, OA participants will be asked to fill out the Chronic Pain Acceptance Questionnaire (CPAQ), the Pain Sensitivity Questionnaire (PSQ), the Perceived Sensitivity to Medicine Scale (PSM), the Five-Factor Personality Index (NEO-FFI), and the Life Orientation Test, Revised version (LOT-R). Healthy participants will be asked a series of questions, most of which are the same as those for the OA and some of which are different and ask about acute painful experiences instead of chronic pain. There are less questions for healthy participants than OA participants, so depending upon how detailed their responses are, healthy participant interviews will last 20-40 minutes. After the interview, healthy participants will be given the PSQ, PSM, NEO-FFI, and LOT-R.

BDI, MPQ, WOMAC, PainDetect, and Pain Catastrophizing Scale Questionnaires will be filled out in an online format during the visit. The online format is almost identical to the hard copy questioner. The CPAQ, PSQ, PSM, NEO-FFI, and LOT-R will be hard copies and will be kept in partipant's study folders. No names will be used, subject ID will be used instead. Moreover, access to the online questionnaires and responses will be password protected and under the


NU IRB Approved
through «ExpireDate»

control of the research coordinator. The patient identification, subject randomization, and treatment monitoring, including safety assessment, will be done under direct supervision of Dr. Thomas Schnitzer (MD, PhD). Study visits will be scheduled as indicated:

**PART ONE:**

Visit 1: (week 0) screening, 15 minute MRI scanning session, and then initiation of placebo

Visit 2: (week 2) evaluate clinical status, collect empty bottles and complete a 10 minute single MRI scan.

For Part one participants will receive compensation for completing visits 1 and 2.Compensation will be given to participants either at the end of each visit (if the amount is $50 or under) or in the form of a check a few weeks later (if the amount is greater than $50). Compensation is as follows:

Visit 1 (Scan 1): $30.

Visit 2 (Scan 2): $30.

**PART TWO:**

Visit 1: Pre-Screening: Medical history, physical exam, questionnaires

Visit 2: Screening: blood test, pregnancy test for women of childbearing potential, MRI scan

Visit 3: Randomization (within 2 weeks of screening): randomization and initiation of study drug/placebo and rescue medications (30 mg/day)

Visit 4: Interim Visit (week 1 ± 3 days): evaluation of clinical status, increase dose of duloxetine/placebo to 60 mg/day

Visit 5: Interim Visit (week 6 ± 7 days): evaluation of clinical status

Visit 6: Final Interim Visit (week 16 ± 14 days): evaluation of clinical status, MRI scan, begin tapering the dose study drug/placebo over a 1 to 2 week period, collect empty bottles and unused rescue medication

Follow-up: Phone Call (week 18 at maximum): evaluation of clinical status, verify that the study drug/placebo was successfully stopped

Optional Visit: Interview (week 20 and beyond): ask questions about mood, pain, and health history, questionnaires

OA participants will receive compensation for completing visits 1, 2, 3, 4, 5, 6, and Optional. Healthy controls will receive compensation for visits 2, 6, and Optional. Compensation will be given to participants either at the end of each visit (if the amount is $50 or under) or in the form of a check a few weeks later (if the amount is greater than $50). Compensation is as follows:


NU IRB Approved
through «ExpireDate»

Visit 1 (Pre-Screening): $25 for OA; N/A for healthy controls

Visit 2 (Screening, with MRI): $50 for all participants

Visit 3 (Randomization): $25 for OA; N/A for healthy controls

Visit 4 (Interim): $25 for OA; N/A for healthy controls

Visit 5 (Interim): $25 for OA; N/A for healthy controls

Visit 6 (Final Interim, with MRI): $50 for all participants

Follow-up (Phone Call): $0 for OA; N/A for healthy controls

Optional Visit (Interview): $25 for all participants

In addition, for both phases of the study, all participants will receive additional compensation for transportation and parking (if applicable), and this compensation is available for all visits (1 through 6, and optional if applicable). We will reimburse individuals up to $20 in various travel expenses, such as CTA fairs or driving costs, and we will validate their parking stickers if they park in the garage at 222 E. Huron Street or at 680 N. Lake Shore Drive.

Brain image data collection and analysis is done under Dr. Apkarian's supervision, which will be performed double blinded. Brain scan data will be transferred to Apkarian's lab where the automated brain morphometric analyses are performed. We contrast brain morphology using a 2-way analysis of variance, factor one is group (patients treated with drug, with placebo, and healthy controls), and factor two is repeat measure time of scan. A secondary analysis will involve correlating brain regional changes to pain (VAS score from sf-MPQ) and the other questionnaire outcomes.

***Study Drug***

Cymbalta® (Duloxetine Delayed-Release Capsules) is a selective serotonin and norepinephrine reuptake inhibitor (SSNRI) for oral administration. Its chemical designation is (+)-(*S*)-*N*-methyl-γ-(1-naphthyloxy)-2-thiophenepropylamine hydrochloride. The empirical formula is C18H19NOS•HCl, which corresponds to a molecular weight of 333.88. The structural formula is:

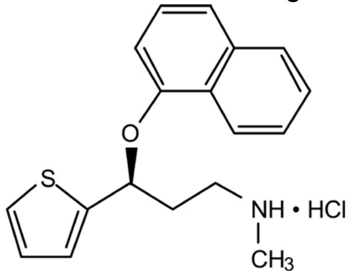


Duloxetine hydrochloride is a white to slightly brownish white solid, which is slightly soluble in water. Each capsule contains enteric-coated pellets of 22.4, 33.7, or 67.3 mg of duloxetine hydrochloride equivalent to 20, 30, or 60 mg of duloxetine, respectively. These enteric-coated pellets are designed to prevent degradation of the drug in the acidic environment of the stomach. Inactive ingredients include FD&C Blue No. 2, gelatin, hypromellose, hydroxypropyl


NU IRB Approved
through «ExpireDate»

methylcellulose acetate succinate, sodium lauryl sulfate, sucrose, sugar spheres, talc, titanium dioxide, and triethyl citrate. The 20 and 60 mg capsules also contain iron oxide yellow.

Although the exact mechanisms of the antidepressant, central pain inhibitory and anxiolytic actions of duloxetine in humans are unknown, these actions are believed to be related to its potentiation of serotonergic and noradrenergic activity in the CNS.

Duloxetine is stored at 25°C (77°F); excursions permitted to 15-30°C (59-86°F). For Chicago Site: Study drug will be maintained in a locked cabinet in a locked room in Abbott Hall. A drug accountability log will be completed each time drug is dispensed or returned and will be maintained by the study coordinator. For Ohio Site: Study drug will be maintained in a locked cabinet in a locked room in NON's office complex. A drug accountability log will be completed each time drug is dispensed or returned and will be maintained by the Deb Scally, the site's study coordinator and Dr. William Bauer, the site's study physician.

Treatment begins at 30 mg once daily for 1 week, to allow patients to adjust to the medication before increasing to 60 mg once daily. Some patients may respond to the starting dose. There is no evidence that doses greater than 60 mg/day confer additional benefit, even in patients who do not respond to a 60 mg dose, and higher doses are associated with a higher rate of adverse reactions. Before discontinuing patients, a gradual reduction in the dose rather than abrupt cessation is recommended. Patients will take 30 mg once daily for 1 week before cessation.

Duloxetine should be swallowed whole and should not be chewed or crushed, nor should the capsule be opened and its contents sprinkled on food or mixed with liquids. All of these might affect the enteric coating. Duloxetine can be given without regard to meals.

Duloxetine's matching placebo pellets are filled into size 1 blue/blue capsugel shells. Overall ingredients: empty gelatin capsules and Nu-Pareil sugar spheres. The matching placebo has a stability of 60 months with minimum allowable storage temperature limits: 15 degrees C/max allowed 25 degrees C. Do not refrigerate or freeze. Route of administration is oral.

Duloxetine is commercially available. Duloxetine and its matching placebo is provided free of charge by the Neuroscience Division of Lilly USA, LLC.

Duloxetine contains a black box warning for Suicidality (suicidal thoughts and/or self-harmful behavior). There is an increased risk of suicidality in children, adolescents, and young adults with major depressive or other psychiatric disorders.

The most common adverse events (happening in more than 5 of 100 people) are:

- Nausea
- Constipation
- Diarrhea
- Decreased appetite
- Dry mouth
- Sweating or night sweats
- Dizziness
- Headache
- Tiredness


NU IRB Approved
through «ExpireDate»

- Weakness
- Drowsiness

Less common adverse events (happening in at least 2 of 100 people) include the following:

- Vomiting
- Stomach and/or abdominal pain
- Increased urination
- Difficulty urinating
- Blurred vision
- Fast or irregular heartbeat
- Weight changes
- Tremors (uncontrollable shaking of a part of the body)
- Changes in sexual desire or ability (see section below)
- Yawning
- Hot flashes
- Acid reflux
- Fever
- Hoarseness or sore throat
- Muscle pain or cramps
- Flu-like symptoms
- Seasonal allergy-like symptoms
- Upper respiratory infection
- Change in taste
- Anxiety or agitation
- Tingling at the fingers or toes, or general differences in sensation
- Rash
- Hives
- Itching

Rare adverse events (happening in 1 of 100 to 1 of 1000 people) include the following:

- Change in blood pressure
- Elevated liver enzymes

Serious adverse events that require immediate medical attention include the following:

- Unusual bruising or bleeding
- Pain in the upper right part of the stomach
- Swelling of the abdomen
- Yellowing of the skin or eyes
- Dark colored urine
- Extreme tiredness or weakness
- Confusion
- A combination of fever, sweating, confusion, and severe muscle stiffness
- Blisters or peeling skin
- Difficulty breathing or swallowing
- Swelling of the face, throat, tongue, lips, eyes hands, feet, ankles, or lower legs

- Worsening of depression-like symptoms
- Mania or hypomania (abnormally elevated mood, arousal, and/or energy-level)
- Withdrawal-like symptoms
- Serotonin syndrome (an adverse event that happens from taking certain combinations of medications; symptoms may involve increased heart rate, shivering, sweating, dilated pupils, intermittent tremor of twitching, changes in your reflexes, stiffness, and/or changes in bowels, blood pressure and body temperature)
- Neuroleptic malignant syndrome (similar to serotonin syndrome mentioned above, with the exception that neuroleptic malignant syndrome often involves a decrease in muscle tone and increased tiredness or weakness)
- Seizures
- Extrapyramidal symptoms (adverse events including extreme restlessness, involuntary movements, and uncontrollable speech)
- Damage to the live
- High blood pressure resulting in organ damage
- Decreased level of sodium in the blood (could result in a coma)
- SIADH (inappropriate secretion of a natural hormone in the body that results in decreased sodium in the blood)
- Glaucoma
- Anaphylaxis (life-threatening allergic reaction)
- Stevens-Johnson syndrome (an adverse event that can lead to blistering of the mouth, palms, and/or soles)
- Erythema multiforme (a skin disorder due to an allergic reaction; may result in multiple circular, raised pink or red lesions)
- Exfoliative dermatitis (loss of the outer layer of skin)

***Randomization and Blinding Design***

For the first part of the study (Phase I), all patients will receive placebo. The design is single blinded; the participants will not know whether they are receiving active drug or placebo. The researcher, however, will know that they are all receiving placebo.

A one-to-one randomization will be used for drug vs. placebo in the second part of the study (phase II). The healthy controls will be matched to the treatment group by age, gender, and education. In addition, the double blind will be maintained throughout all study procedures.

***Study Endpoints***

Primary: Overall neocortical gray matter volume and regional gray matter density in patients treated with Duloxetine and placebo

Secondary: Changes in gray matter volume and regional density measures will be correlated with changes in pain parameters

Exploratory (Optional Visit): Differences in past experience and current expectations, as measured with the open-ended interview, will be


NU IRB Approved
through «ExpireDate»

correlated to treatment response and to differences and/or changes seen in brain parameters

***Study Procedures (more detailed)***:

Study visit procedures (for OA participants):

**PART ONE:**

Visit 1: **Screening/Baseline Scan** (week 0) Medical history, physical examination, patient-reported outcome (PRO) battery of instruments (includes: a general health questionnaire (PHH), the short form of McGill Pain Questionnaire (sf-MPQ), WOMAC, and the Beck Depression Index (BDI)), the Pain detect questionnaire, and the Pain catastrophizing scale(PCS), pregnancy test (if female of child-bearing potential),complete a 15 minute MRI scan, dispense study drug.

Visit 2: **Final visit/Scan2** (week 2) PRO battery, one 10 minute MRI scan, collect information on concomitant medications, collect unused rescue medications and empty study drug bottles

**PART TWO:**

Visit 1: **Pre-Screening**. Medical history, physical examination, patient-reported outcome (PRO) battery of instruments (includes: a general health questionnaire (PHH), the short form of McGill Pain Questionnaire (sf-MPQ), WOMAC, and the Beck Depression Index (BDI)), the Pain detect questionnaire, and the Pain catastrophizing scale (PCS)

Visit 2: **Screening/Baseline Scan**. Basic chemistry panel and complete blood count (CBC), pregnancy test for females of child-bearing potential, MRI scans

Visit 3: **Randomization**. (Week 0) WOMAC, dispense Duloxetine/placebo (at 30mg/day) and rescue medications, collect information on concomitant medications

Visit 4: **Interim Visit.** (Week 1 ± 3 days) Dispense drug/placebo (increase dose of duloxetine/placebo to 60 mg/day), WOMAC, collect AEs and concomitant medication information

Visit 5: **Interim Visit.** (Week 6 ± 7 days) Dispense drug/placebo (same dose as in Visit 3), WOMAC,BDI, collect AEs and concomitant medication information

Visit 6: **Interim Visit**. (Week 16 ± 14 days) Dispense drug/placebo (begin to gradually reduce dose via a down-titration of 30mg for 1 week), MRI scans, PRO battery, collect AEs and concomitant medication information; collect unused rescue medications and empty duloxetine/placebo bottles

Follow-up: **Phone call**. (Week 18 maximum) Collect AEs and concomitant medication information, verify that participant has successfully stopped the drug/placebo



**NU IRB Approved through «ExpireDate»**

Optional: **Interview** (Week 20 and beyond): Ask a series of open-ended questions about participants' experiences with pain, overall mood and personality, and medical history; give questionnaires about personality and pain/medical beliefs and expectations

Control participants will only complete visits 2 and 6. More specifically, they will only complete the PHH (once during screening) and the BDI (at all visits). Urine dipstick pregnancy tests will only be done in healthy controls if the participant is a woman of child-bearing potential. No blood will be drawn. Control participants also have the have the choice of completing an optional visit at the very end of the study that involves answering questions in an open-ended interview and completing a set of new questionnaires that supplement this interview.

For part two of the study, high-resolution anatomic brain images and functional brain scans are collected before and after four-months of treatment with duloxetine in 30 OA patients, in 30 OA patients who receive placebo, and in 25 healthy controls. Brain morphology is contrasted between these two time points, across the three groups, for overall neocortical gray matter volume and regional gray matter density changes. Pain relief, based on questionnaire outcomes and the MPQ's VAS, are then used as covariates in both patient groups to identify the effect size of magnitude of pain relief on restoration of brain grey matter. The procedures follow exactly our publications on the topic (Geha et al., Neuron. 2008; 60: 570. Apkarian et al., J. Neuroscience 24:10410, 2004).

***Recruitment, Selection, and Discontinuation of Subjects***

<u>Study Population:</u>

Patients with chronic (>1year) symptoms of knee osteoarthritis

<u>Recruitment and Enrollment Design</u>

Participants will be recruited from an osteoarthritis patient database available through two sites. The first site is the Rehabilitation Institute of Chicago (RIC), which the study doctor (Dr. Thomas Schnitzer) has access to andthe second site is the Northern Ohio Neuroscience, LLC, which the study doctor (Dr. William Bauer) has access to. They will be contacted via telephone to see if they are interested, and if so, to see if they qualify for the screening visit. All participants will complete a screening visit with either Dr. Schnitzer or Dr. Bauer to determine if they are eligible to continue in the study. Eligibility will be based on the inclusion and exclusion criteria stated below, and these criteria will be measured through a physical exam, blood tests (to screen for abnormal chemistry panels and complete blood counts, as well as pregnancy if applicable), answers participants give in their questionnaires and MRI screening forms, as well as those regarding their medical history and current medications.

Control participants will undergo a similar screening visit, except that they do not need to complete a physical exam or blood tests. If the participant is a woman of child-bearing age, then she must give urine to be used for a urine dipstick pregnancy test).

Participants will be given a written, informed consent form during the screening visit, and if they are eligible and want to participate, they will be invited to come back for subsequent visits. Note that healthy controls will only complete two visits (screening/baseline scan and the final visit involving an MRI scanning session).



**NU IRB Approved through «ExpireDate»**

Number of Subjects:

* 80 participants with chronic osteoarthritis in their knee (20 in phase I and 60 in phase II. The 20 in phase I will all receive placebo. In phase II, 30 will receive the study drug; 30 will receive placebo)

* 25 healthy controls

Inclusion and exclusion criteria:

***Major inclusion criteria include:***

* Age: 45-80 years

* ACR criteria for OA including Kellgren-Lawrence radiographic OA grades II-IV

* VAS pain score ≥5/10 within 48 hrs of the phone screen and visit 1 (Screening)

* Knee OA for a minimum of 12 months

* Need for daily pain medication to manage symptoms of OA

***Major exclusion criteria include:***

* Currently taking MAO inhibitors or any centrally acting drug for analgesia, depression

* Narrow angle glaucoma

* Uncontrolled hypertension

* Co-existing inflammatory arthritis, fibromyalgia or other chronic pain state.

* If a female, pregnant, trying to become pregnant, or lactating

* Major depressive disorder

* Substantial alcohol use or history of significant liver disease

* Use of MAO inhibitors, triptans, serotonin precursors (tryptophan)

* Use of potent CYP1A2 inhibitors, Thioridazine, and anti-depressants

* Diabetes, type 1 or type 2

* Condition in which the Investigator believes would interfere with the subject's ability to comply with study instructions, or might confound the interpretation of the study results or put the subject at undue risk

* MRI safety necessitates the exclusion of subjects having one or more of the following:

- Metal fragments in the eye or face, or having worked previously in the metal industry
- Implantation of any electronic devices such as (but not limited to) cardiac pacemakers, cardiac, defibrillators, and cochlear implants or nerve stimulators.
- Surgery on the blood vessels of the brain
- Claustrophobia (fear of enclosed places)
- Piercings or tattoos
- More than 250 lbs in weight

- Obvious brain abnormalities

### *Efficacy Assessment*

Efficacy will be assessed primarily through the utilization of the WOMAC indices, specifically those regarding changes in pain, functionality, and stiffness in the knee. In addition, changes in VAS scores (from the sf-MPQ) could also be used to measure the drug's efficacy with regard to pain relief. The BDI scores could also be used to measure emotional or cognitive changes associated with the active drug, and thus act as an assessment tool in this regard.

### *Safety Assessment*

The main ethical considerations are adverse effects of the active drug and subject safety for MRI scans. Regarding the first consideration, we will monitor adverse effects of the study drug by talking with participants at their clinical visits, collecting/recording any symptoms they mention, and reviewing participants' answers on the PRO battery questionnaires.

As for the second consideration, MRI safety is routinely implemented at Northwestern University brain imaging center, and there is an MRI screening form that must be completed and reviewed by trained personnel before every scan. If unrestrained iron or steel objects are accidentally brought near the MRI magnet, they can be pulled very quickly toward the magnet and can strike people in or near the magnet. Such an event is very unlikely, because precautions are taken to prevent such objects from being brought near the magnet. Participants are screened for iron or steel implants or clips from surgery, or metallic objects, such as shrapnel or metal slivers in their bodies, are excluded from the study if present. Dental fillings do not present a hazard.

A third issue is participant identity protection. All analyses and report of results will be based on using anonymized data. In addition, files of all participants will be confidential and will be available only to authorized study personnel.

### *Statistical Analysis*

As soon as participants' MRIs are collected they are transferred to Apkarian's lab where again we check for quality. Final data analysis cannot begin until all the repeat brain scans are collected, where we then follow procedures that we have used in the past. The correlative analyses require also getting all the pain questionnaire and drug use outcomes at study completion.

All participants' scans will be run through a quality control pipeline. Those participants whose scans are returned by the pipeline as poor in quality (e.g, they may have a low signal-to-noise ratio or too much movement), and verified to be so by qualified personnel, will *not* be included in data analysis concerning how the brain changes under the influence of the study drug. However, these participants' questionnaire data, medical history, and pain reportings may still be used in data analysis concerning drug efficacy.

Brain morphometry analysis follows our published procedures (Geha et al., Neuron. 2008; 60: 570. Apkarian et al., J. Neuroscience 24:10410, 2004). Briefly, we use three approaches to compare brain anatomy between groups (primary hypothesis) and correlate brain anatomical parameters with pain relief in the treatment arm (secondary hypothesis). For all subjects we



**NU IRB Approved through «ExpireDate»**

calculate whole-brain gray matter volume for the neocortex, normalized by skull size, and corrected for sex and age confounds. In the first approach, this value is then contrasted between treatment and placebo groups, which test the primary hypothesis for the whole-brain. In a second analysis, we perform voxel-based morphometry (VBM) where we calculate gray matter density for each voxel of gray matter (3 $mm^3$, about 20,000 voxels/brain). The VBM results are corrected for whole-brain volume, age and sex, and then contrasted between the groups. This contrast indicates what specific brain regions have changed in gray matter density and in what direction. In the third approach, we subdivide the neocortical gray matter into 80 morphological sections (Brodmann areas), correct them for age, sex, and whole brain volume, and contrast them between the two groups.

For the secondary hypotheses, the whole-brain volume, specific brain regions that show a change in VBM and Brodmann areas that show changes between groups are then regressed in the treatment group with pain measures. The sf-MPQ will be used to derive scalars for sensory and affective dimensions of osteoarthritis pain. Anxiety and depression traits will be determined by the BDI questionnaire, and intensity of pain will be assessed on a visual analog scale (0 - no pain, 10 - maximum imaginable pain), which is also part of the sf-MPQ. These characteristics will be related to brain morphometry and atrophy either by linear correlations or in multiple linear regression models with stepwise elimination. We do not expect to see a significant change with placebo treatment. However, we will regress the placebo pain measures with the brain regions identified as changing. If our assumption is correct, then this approach will distinguish between the biological effects of the treatment from the psychological (placebo) effects.

Sample Size Considerations:

Effect size: Given that the design is a within subject repeat measures analysis of variance, the overall variance should be about half of that we had originally anticipated. In fact, two recent studies were able to show significant restoration of grey matter in n=10 patients with such a design. However, in those two studies the pain relief was complete for many months. Here we expect a smaller decrease in pain (between 10-20mm on VAS scale from baseline) and, as a result, we have increased the sample size of the population treated with drug three times. Moreover, assuming that pain relief will be less in the placebo group, inclusion of this arm into the study should strengthen the power of the study. The healthy normal subjects tested at two equivalent time points is necessary to rule out that the observed results are scanner artifacts. In the normal subjects we specifically test that in the same time period and using the same scanner we observe no significant, or minimal increased atrophy (0.75 cc increase in atrophy, in the direction opposite of what we expect to see with drug treatment). Moreover, since we already have results regarding brain regions where atrophy is observed in OA, we can perform brain region-specific directed contrasts between the groups, if necessary. Overall, this design should give us enough power to test the primary and secondary hypotheses. It should be noted, however, that this is an exploratory study, and there is no statistical justification to our proposed sample size.

**Potential Problems or Pitfalls:**

The Radiology department performs routine quality control studies on the MR scanner, to insure that signal-to-noise ratio and contrast-to-noise ratio for functional scans are stable. If we detect drifts in these parameters, we would stop recruitment and pause scanning visits to attend to these problems. Such occurrences are rare, about once a year, and are usually handled by


NU IRB Approved
through «ExpireDate»

Radiology in a routine fashion. There are a number of small technical details that are necessary to function properly for the fMRI studies. These are routinely tested prior to every fMRI scan, and inevitably occasionally fail. For this reason we have a complete second back-up system of the equipment that can replace any of the malfunctioning parts. Thus, we can almost always perform a scheduled scanning session for participants, and repair the damage subsequently. We expect that the scanner might fail in about 5% of the scans maximum, which may then need to be rescheduled, usually within 24 hours.

**Anticipated Results:**

We expect to demonstrate that overall mean neocortical gray matter volume and regional gray matter density is increased from pre- to post-treatment in patients treated with duloxetine but not in placebo-treated patients.

For the secondary hypotheses, we will correlate gray matter volume and regional density measures with pain parameters, and we expect to demonstrate a correlation between the extent of pain relief and the change in magnitude of brain atrophy.



**NU IRB Approved through «ExpireDate»**
