# Supplementary material for: Brain Connectivity Predicts Placebo Response across Chronic Pain Clinical Trials
Source: PLoS Biol. 2016 Oct 27;14(10):e1002570. doi: 10.1371/journal.pbio.1002570 (PMC5082893; doi:10.1371/journal.pbio.1002570)
Supplement: S2 Study Protocol — (PDF) [file pbio.1002570.s006.pdf]

Epidemiology of OA Pain

Principal Investigator: Thomas J. Schnitzer, MD, PhD

Office Address: 710 N. Lake Shore Drive, Room 1020
Chicago, IL 60611

Study sites: 680 N. Lake Shore Drive, Suite 1410
Chicago, IL 60611

710 N. Lake Shore Drive, Rooms 1020, 1014
Chicago, IL 60611

Co-Investigators: Jennifer Duffecy, PhD
Mark Begale

Project Overview/Summary

Chronic pain, by definition, persists over long periods of time, but both sufferers of pain and their caregivers are aware that pain levels can fluctuate greatly over time. Being able to define pain variability and its underlying causes would be an important advance in our understanding of chronic pain, and would in turn lead to better approaches to the treatment of chronic pain. In this study, we propose to evaluate in 160 people with chronic pain of the knee due to osteoarthritis over 3 months, collecting data regarding magnitude of pain, physical activity level, and medication status twice daily during this period of time. We then will assess the variability in pain levels over time, the frequency of "flares" of OA pain and their relationship to physical activity, medications, and clinical descriptors of the population, e.g., age, gender, BMI. Additionally, evaluations will be made regarding temporal patterns of OA knee pain as well as assessment of subgroups of patients with the overall group being studied.

Background:

Osteoarthritis (OA) is a chronic pain state that has been associated with pathologic changes involving all the different tissues of the joint, including articular cartilage, underlying bone, surrounding synovium, and peri-articular structures such as muscle and tendon. The etiology of pain in OA is not known, other than it not arising from cartilage itself which is aneural. There has been a growing appreciation of the importance of input from peripheral nociceptors, though what the instigating stimuli are is subject of discussion. It is likely that there will be no single pathway for joint pain, but rather many different mechanisms may give rise to the clinical phenotype. There is evidence for changes in the subarticular bone, seen best by MRI and called "bone edema", and these changes have correlated with symptomatic knee OA. Inflammatory changes are seen in synovium and various markers of inflammation may be seen in joint fluid and serum, particularly in long-standing OA. Drugs known to be effective in the treatment of OA appear to be able to modulate the pain response either by interfering peripherally, e.g., blocking sensitization of peripheral nociceptors (NSAIDs), or centrally, e.g., enhancing

descending inhibitory pathways (SNRIs). The demonstration that topical NSAIDs are effective in the management of the pain of knee OA, and their subsequent marketing approval by the FDA, supports that topical application of analgesic agents is capable of effecting a clinically meaningful response in this pain condition.

Despite what we know about the mechanisms of pain in OA, there have been no published reports describing the variability in pain response from day to day in patients with OA. "Flares" of OA are said to be common, yet the true frequency of these "flares" and even what constitutes a "flare" has not been well defined. A recent publication that has just appeared on the web (not yet in print) suggests that in a clinical trial setting, knee OA "flares" may occur at the rate of approximately 1/month in individuals taking medications on an as-needed basis and possibly less often if they are taking their medications on a regular basis. This study suffers from significant methodological limitations but does allow for an estimate of the frequency of "flares" as defined by the authors. Clearly, having a better understanding of how pain levels fluctuate, both during the day and from day to day, and how these levels of pain correlate to activity levels and perhaps other variables in people with OA would be important in order to consider recommendations regarding management of the pain. This knowledge would permit an informed approach to time and frequency of administration of treatment and even to the type of treatment that should be considered. Such information is lacking and gaining this information is the goal of the current study.

Primary/Secondary Objectives:

The primary objective of this study is to characterize and describe the variability in daily pain over an extended period of time in people with knee OA. Secondary objectives are to determine if pain correlates with physical activity, medication usage, or any of the baseline descriptors of the population being studied, and whether changes in pain that are observed themselves will lead to changes in these same parameters, e.g., physical activity, medication usage.

Research Design:

This is a longitudinal study of individuals with knee OA who report knee pain most days of the week. There will be no requirement for mediation usage as long as individuals are experiencing a frequency of knee pain that meets our inclusion criteria (see below). A total of 160 subjects will be recruited and followed for 3 months during which they will be asked to follow their usual activities. At baseline and at each monthly clinic visit participants will complete a number of standard validated instruments to assess pain, function and other attributes. Up to thrice-daily assessment of pain level, function and medication usage will be collected by interactive cellphone-based telephony. Participants will be encouraged to engage in their normal range of activities and continue to take medications as needed to manage their OA pain. No medications will be given in the study and none will be prohibited from being started during the course of the participants' involvement in the trial.

Inclusion criteria:
1. Men and women, age 40 years and above
2. Knee pain most days of the week for the past month

3. Diagnosis of knee OA
4. Meet ACR criteria for knee OA
5. No significant limitations in physical activity
6. Own and able to use a cellular phone capable of text messaging
7. Able to return for all clinic visits
8. Able to read and understand the informed consent document

Exclusion criteria:

1. Use of a walker to ambulate or inability to ambulate (use of cane is allowed)
2. Other forms of arthritis
3. Other major causes of pain that could be expected to interfere with assessment of pain during this trial, e.g., recurrent migraine, back pain, fibromyalgia
4. Scheduled for and likely to need joint replacement surgery in the next 3 months
5. Any medical condition that in the judgment of the investigator would make the participant not suitable for the study
6. Living in a long-term care facility

Procedures:

Screening/Baseline Visit (Day 1):

Participants will be seen in an examination room and told about the study in detail and then given the informed consent document to read. They will have their questions answered and then be asked to sign the document before any procedures are done. Consenting will be done by the study coordinator and the PI will be available to answer questions. Those individuals interested in participating will then have a medical history obtained and undergo a physical examination. If they are found to qualify for the study, they will be asked to complete a battery of instruments to assess their pain level and functional status. They will then be instructed in the use of the cell phone response system and will be asked to successfully complete an initial response in the office.

Day 2: Phone follow-up.

All participants will be contacted to assure that they are using the phone response system appropriately and answer any outstanding questions. If there are problems identified, further telephone calls will take place on subsequent days until the participant is using the system appropriately.

Week 2 visit:

Participants will return to the clinic and be asked to complete the same battery of pain and level of function assessments as at baseline. Assessment of their appropriate use of the cell phone response system will be checked and discussed with the participant; additional instruction will be provided as needed. Information about use of concomitant medications and change in ongoing medications will be obtained as will any adverse events that have occurred.

Week 6 visit:

Identical procedures to Week 2 visit.

Week 12 visit:

Participants will complete the full battery of pain and level of function assessments. Information about use of concomitant medications and change in ongoing medications will be obtained as will any adverse events that have occurred.

Outcome Measures:
NRS Pain Scale (Farrar 2001): Pain intensity is measured on an 11 point numerical rating scale, where 0 = no pain and 10 = worst possible pain. This scale has been widely used and validated for many different pain states and clinical significance of changes determined.
Western Ontario and McMaster Universities Arthritis Index (WOMAC®; Bellamy 1982): is a well-validated OA-specific instrument that evaluates 3 domains associated with OA: pain (5 questions), function (17 questions) and stiffness (2 questions). It has been used repeatedly in knee and hip OA studies, including assessing outcome after joint replacement surgery.
Patient Global Impression of Change (Farrar et al. 2001): assesses overall disease status as well as response to treatment using a 5-point categorical scale with descriptors of overall disease and response to therapy.
PQAS (Pain Quality Assessment Scale: Jensen MP, 2006) is a well-validated instrument that includes 20 items to assess different qualities of pain perception and has been utilized in studies of neuropathic and non-neuropathic pain states.
SF-12 (Gandek et al. 1998): is a well-validated health related quality of life instrument, which measures multiple dimensions of the pain experience. The long form (SF-36) is very frequently used in pain studies (Rowbotham et al. 1998), and the SF-12 shows good validity with the longer version of the instrument (Gandek et al. 1998).
PIRS (Pittsburgh Insomnia Rating Scale: Moul et al. 2002). An adapted version of this questionnaire will be used to evaluate changes over a single night.

Descriptive and classification outcomes
Personal Health History (PHH): This general questionnaire will assess demographic information (gender, age, marital/relationship status, race/ethnicity, and education), pain history (descriptions, diagnostic testing history, and treatment history), general health history (other medical/surgical history, concurrent medications, health behaviors, a general symptom checklist, and health-care utilization), and work history. The entire questionnaire will be completed at the screening visit. History and physical examination will be done to confirm diagnosis, inclusion and exclusion criteria and assess overall safety.

Participant Compensation:
Participants will receive up to $150 for taking part in this research study. $25 will be paid in cash at the time of the first visit, $35 at the time of the second visit, and $45 at each of the last two visits. The increase in payments is to help pay for the use of their cell phone during the course of the study. Payments will not be prorated but paid as noted above.

Participant Privacy:
All participant research-related information will be deidentified. All personal information will be kept in a locked, secure location. All physical materials are kept in locked cabinets and locked rooms with access only to study personnel. All digital data will be password protected. All identifiable data will be maintained for at least 2 years after the end of the trial or after publication of results. Storage will be off-site at our storage location, Reebie.

Risk and Benefit:
Greater awareness of participants' level of pain and function may be considered both a risk and benefit accruing from involvement in this minimal-risk study; there may be emotional

discomfort in answering questions involved in the study. Participants will be told that they may become more aware of their pain and function based on answering these questions, and that if this is bothersome they should call the study coordinator to inform us and that they are free to not answer questions or discontinue the study at any time.

Sample Size:
As this is a descriptive study without a formal comparative endpoint, no prespecified total number of participants can be determined. It is anticipated that 160 subjects followed for 3 months should provide an adequate dataset to be able to evaluate the frequency and severity of pain flares that occur during daily activity and ascertain further features characteristic of OA pain, e.g., association with activity, with medication use and change of medications, temporal variations in pain. Moreover, this sample size will be sufficient to estimate population parameters with reasonably small standard errors, and is consistent in size with many other treatment studies. It is not known how many people may not finish the complete 3 months and therefore we will plan on enrolling up to 160 participants if necessary.

Statistical Analysis:
Exploratory analyses will be conducted into the correlation of absolute pain levels as well as changes in pain ("flares") with a variety of clinical and demographic parameters that will be collected, including age, gender, BMI, physical function, medication use, change in medication, etc. We will use 95% confidence intervals to test statistical significance of correlation coefficients, but all statistical analyses will include graphical analyses (e.g., scatterplots of bivariate relationships) to inspect the shape of relationships. The temporal pattern of pain will be assessed (diurnal, weekly, monthly variability) and modeled, which will allow us to investigate how covariates influence changes in pain over time. When needed, principal component analysis and associated factor analytic techniques will be used to reduce variables into meaningful clusters.

Publication Plan:
We anticipate submitting one or more abstracts of these data to clinical pain or rheumatology meetings, e.g., APS and/or ACR, within 3 months of completing data collection, and then submitting at least one final manuscript within another 3 months to either a pain journal (Clinical Journal of Pain, Journal of Pain) or a rheumatology journal (Arthritis & Rheumatism, Arthritis Care & Research).
