# Supplementary material for: Brain Connectivity Predicts Placebo Response across Chronic Pain Clinical Trials
Source: PLoS Biol. 2016 Oct 27;14(10):e1002570. doi: 10.1371/journal.pbio.1002570 (PMC5082893; doi:10.1371/journal.pbio.1002570)
Supplement: S1 Table — Values are shown as mean and 1 SE (in parenthesis). Duration = duration of OA knee pain, in years; MQS, Medication Quantification Scale = medication use at time of entry into study. (DOCX) [file pbio.1002570.s007.docx]

| **Group** | **Study 1 (placebo)** | | **p value** | **Study 2 (placebo)** | | **p value** | **Study 2 (Duloxetine)** | | **p value** | **Controls** |
| --- | --- | --- | --- | --- | --- | --- | --- | --- | --- | --- |
|  |  |  |  |  |  |  |  |  |  |  |
| **Responder** | Yes | No | — | Yes | No | — | Yes | No | — | — |
| **Gender** | 5F/3M | 4F/5M | — | 7F/4M | 5F/5M | — | 5F/3M | 5F/6M | — | 10F/10M |
| **Age** | 55.9 (1.5) | 57.8 (2.3) | 0.51 | 54.7 (3.0) | 62.0 (2.6) | 0.08 | 57.6 (0.8) | 60.3 (1.7) | 0.17 | 57.9 (1.5) |
| **Duration** | 11.4 (3.6) | 13.3 (3.8) | 0.72 | 10.2 (2.8) | 12.7 (3.4) | 0.57 | 7.3 (2.4) | 11.6 (3.1) | 0.23 | — |
| **BDI** | 3.4 (1.5) | 5.2 (1.4) | 0.39 | 2.7 (1.2) | 10.5 (3.6) | 0.07 | 3.8 (1.6) | 7.6 (3.3) | 0.31 | 2.5 (2.2) |
| **PCS** | 25.0 (5.4) | 16.9 (4.5) | 0.27 | 9.0 (2.2) | 24.5 (4.2) | 0.006 | 5.4 (0.9) | 18.3 (4.8) | 0.03 | — |
| **MQS** | 7.1 (0.7) | 7.0 (0.6) | 0.91 | 4.7 (2.2) | 10.0 (3.8) | 0.25 | 5.1 (2.5) | 10.6 (4.8) | 0.32 | — |
