# Supplementary material for: Brain Connectivity Predicts Placebo Response across Chronic Pain Clinical Trials
Source: PLoS Biol. 2016 Oct 27;14(10):e1002570. doi: 10.1371/journal.pbio.1002570 (PMC5082893; doi:10.1371/journal.pbio.1002570)
Supplement: S2 Table — Values are shown as mean and 1 SE (in parenthesis). Duration = duration of OA knee pain, which was only available in 50% of participants; VAS = knee OA pain. (DOCX) [file pbio.1002570.s008.docx]

| **Group** | **Study 3 (no-treatment) n = 42** | **Study 3 (no-treatment) n = 20** |
| --- | --- | --- |
|  |  |  |
| **Gender** | 25F/17M | 10F/10M |
| **Age** | 56.3 (1.0) | 57.1 (1.5) |
| **Duration** | >10 years | >10 years |
| **VAS (baseline)** | 5.6 (0.35) | 6.4 (0.41) |
| **VAS (2 weeks)** | 5.2 (0.32) | 5.9 (0.41) |
| **VAS (3 months)** | 4.7 (0.32) | 5.7 (0.41) |
