# Supplementary material for: Brain Connectivity Predicts Placebo Response across Chronic Pain Clinical Trials
Source: PLoS Biol. 2016 Oct 27;14(10):e1002570. doi: 10.1371/journal.pbio.1002570 (PMC5082893; doi:10.1371/journal.pbio.1002570)
Supplement: S3 Table — The degree counts from the r-MFG region extracted from scans for study 1 and study 2 (placebo-treated groups) were correlated with the knee pain values (VAS and WOMAC) obtained before and after the placebo treatment. No correlation was observed with VAS before treatment for both groups, while VAS after treatment significantly correlated with r-MFG degree count. A similar, but not as robust, pattern was observed for WOMAC as well. Data are shown as R-values (p-values are shown in parentheses, with significant correlations in bold). This analysis was performed to test the extent to which r-MFG counts may be reflecting regression to the mean. The obtained results are suggestive (but do not fully rule out) that r-MFG counts reflect the placebo response rather than regression to the mean. (DOCX) [file pbio.1002570.s009.docx]

| Degree counts | Correlation with VAS before (p) | Correlation with VAS after (p) | Correlation with WOMAC before (p) | Correlation with WOMAC after (p) |
| --- | --- | --- | --- | --- |
| r-MFG study 1 | 0.37 (0.14) | **-0.53 (0.03)** | 0.20 (0.45) | -0.38 (0.14) |
| r-MFG study 2 placebo | 0.03 (0.99) | **-0.58 (0.01)** | -0.41 (0.07) | **-0.57 (0.01)** |
