# Supplementary material for: Brain Connectivity Predicts Placebo Response across Chronic Pain Clinical Trials
Source: PLoS Biol. 2016 Oct 27;14(10):e1002570. doi: 10.1371/journal.pbio.1002570 (PMC5082893; doi:10.1371/journal.pbio.1002570)
Supplement: S1 Text — (DOCX) [file pbio.1002570.s010.docx]

**Supporting information to:**

**Brain connectivity predicts placebo response across**

**chronic pain clinical trials**

Pascal Tétreault^1^, Ali Mansour^1^, Etienne Vachon-Presseau^1^, Thomas J. Schnitzer^2,3^, A. Vania Apkarian^1,2,4,*^ and Marwan N. Baliki^1,*^

Departments of ^1^Physiology, ^2^Physical Medicine and Rehabilitation, ^3^Internal Medicine and ^4^Anesthesia, Northwestern University, Feinberg School of Medicine, Chicago, Illinois 60611, USA.

*Correspondence to A. Vania Apkarian, email: [a-apkarian@northwestern.edu](mailto:a-apkarian@northwestern.edu).

*Correspondence to Marwan N. Baliki, email: [m-baliki@northwestern.edu](mailto:m-baliki@northwestern.edu).

**Inclusion/exclusion criteria, 1 supplementary figure, 3 supplementary tables.**

**Supplementary methods:**

Full inclusion and exclusion list for all 3 studies was as following:

**Inclusion Criteria:**

- Age: 45-80 years
- ACR criteria for OA including Kellgren-Lawrence radiographic OA grades II-IV
- VAS pain score >5/10 within 48 hrs of the phone screen and visit 1 (Screening)
- Knee OA for a minimum of 12 months
- Need for daily pain medication to manage symptoms of OA

**Exclusion Criteria:**

- Currently taking MAO inhibitors or any centrally acting drug for analgesia, depression
- Narrow angle glaucoma
- Uncontrolled hypertension
- Co-existing inflammatory arthritis, fibromyalgia or other chronic pain state.
- If a female, pregnant, trying to become pregnant, or lactating
- Major depressive disorder
- Substantial alcohol use or history of significant liver disease
- Use of MAO inhibitors, triptans, serotonin precursors (tryptophan)
- Use of potent CYP1A2 inhibitors, Thioridazine, and anti-depressants
- Diabetes, type 1 or type 2
- Condition in which the Investigator believes would interfere with the subject's ability to comply with study instructions, or might confound the interpretation of the study results or put the subject at undue risk
- **MRI safety necessitates the exclusion of subjects having one or more of the following:**
  - Metal fragments in the eye or face, or having worked previously in the metal industry
  - Implantation of any electronic devices such as (but not limited to) cardiac pacemakers, cardiac, defibrillators, and cochlear implants or nerve stimulators.
  - Surgery on the blood vessels of the brain
  - Claustrophobia (fear of enclosed places)
  - Piercings or tattoos
  - More than 250 lbs in weight
  - Obvious brain abnormalities

More details on study 1 can also be found in **Study1-2_protocol.pdf** and on clinicaltrials.gov with the following accession number: NCT02903238.

More details on study 2 can also be found in **Study1-2_protocol.pdf** and on clinicaltrials.gov with the following accession number: NCT01558700.

More details on study 3 can also be found in **Study3_protocol.pdf**
